# Combined light and electron microscopy (CLEM) to quantify methamphetamine-induced alpha-synuclein-related pathology

**DOI:** 10.1007/s00702-024-02741-x

**Published:** 2024-02-17

**Authors:** Michela Ferrucci, Paola Lenzi, Gloria Lazzeri, Carla L. Busceti, Alessandro Frati, Stefano Puglisi-Allegra, Francesco Fornai

**Affiliations:** 1 Department of Translational Research and New Technologies in Medicine and Surgery, University of Pisa, Via Roma 55, 56126 Pisa, Italy; 2 Istituto di Ricovero e Cura a Carattere Scientifico (I.R.C.C.S.) Neuromed, Via Atinense 18, 86077 Pozzilli, Italy; 3 Neurosurgery Division, Human Neurosciences Department, Sapienza University, 00135 Rome, Italy

**Keywords:** Neuronal inclusions, Parkinson’s disease, Addicted brain, Autophagy, Poly-ubiquitin, p62

## Abstract

Methamphetamine (METH) produces a cytopathology, which is rather specific within catecholamine neurons both in vitro and ex vivo, in animal models and chronic METH abusers. This led some authors to postulate a sort of parallelism between METH cytopathology and cell damage in Parkinson’s disease (PD). In fact, METH increases and aggregates alpha-syn proto-fibrils along with producing spreading of alpha-syn. Although alpha-syn is considered to be the major component of aggregates and inclusions developing within diseased catecholamine neurons including classic Lewy body (LB), at present, no study provided a quantitative assessment of this protein in situ, neither following METH nor in LB occurring in PD. Similarly, no study addressed the quantitative comparison between occurrence of alpha-syn and other key proteins and no investigation measured the protein compared with non-protein structure within catecholamine cytopathology. Therefore, the present study addresses these issues using an oversimplified model consisting of a catecholamine cell line where the novel approach of combined light and electron microscopy (CLEM) was used measuring the amount of alpha-syn, which is lower compared with p62 or poly-ubiquitin within pathological cell domains. The scenario provided by electron microscopy reveals unexpected findings, which are similar to those recently described in the pathology of PD featuring packing of autophagosome-like vesicles and key proteins shuttling autophagy substrates. Remarkably, small seed-like areas, densely packed with p62 molecules attached to poly-ubiquitin within wide vesicular domains occurred. The present data shed new light about quantitative morphometry of catecholamine cell damage in PD and within the addicted brain.

**Supplementary Information:**

The online version contains supplementary material available at 10.1007/s00702-024-02741-x.

## Introduction

In recent years, a significant progress occurred concerning the nature of alpha-synuclein (alpha-syn)-related cytopathology, encompassing small protein aggregates and frank neuronal inclusions, which develop within damaged catecholamine neurons. This mostly applies to Lewy Bodies (LB), which characterize neurodegeneration in Parkinson’s disease (PD) and Lewy Body Dementia (LBD). It is now established that alpha-syn-related cytopathology and inclusions do occur within catecholamine cells also following methamphetamine (METH) exposure (Vincent and Shukla 2023) in rodents (Fornai et al. 2004) and humans (Quan et al. 2005; Wu et al. 2021). This neuropathology observed in chronic METH abusers resembles to what observed in PD patients (Vincent and Shukla 2023). In detail, METH-induced neurodegeneration involves aggregation of alpha-syn proto-fibrils within catecholamine neurons and drives these neurons to make them more vulnerable to degeneration as recognized in Parkinson's disease (Vincent and Shukla 2023). In fact, wide cytosolic areas during parkinsonism and following METH exposure similarly stain for alpha-syn, poly-ubiquitin, and other antigens related to the autophagolysosomal pathways (Fornai et al. 2004; Forno 1996; Quan et al. 2005; Shimura et al. 1999; Spillantini et al. 1997; Wulf et al. 2022). This extends to cell-to-cell propagation of alpha-syn following METH, and deleterious effects of alpha-syn expression in METH-induced neurodegeneration (Ding et al. 2020; Meng et al. 2020; Wu et al. 2021). This is in line with the involvement of autophagy and autophagolysosomes in the damage of catecholamine cells (Anglade et al. 1997; Chandra et al. 2004; Ferrucci et al. 2008; Fornai et al. 2003, 2005; Larsen et al. 2002; Sato et al. 2018). An intense debate is ongoing concerning the intimate structure of alpha-syn-related cytopathology. In fact, the nature of neuronal inclusions is seminal to comprehend the neurobiology of disease and to dissect, which specific step needs to be targeted to plan disease-modifying therapies [either in neurodegeneration or in the addicted brain (Iacovelli et al. 2006; Mahul-Mellier et al. 2020)]. The task of deciphering the structure of neuronal inclusions and molecular components building up cytopathology within catecholamine cells would require a quantitative analysis of specific components. Unfortunately, a quantitative analysis in situ is quite complex and it was never carried out. Thus, inclusions are identified only by qualitative approaches, based on immuno-histochemistry or plain electron microscopy. To our knowledge, no quantitative assessment comparing specific proteins or non-protein components was carried out in situ. Such a measurement requires to be established in situ to avoid the bias of measuring dispersed cytosolic areas or nuclear components. Such an in situ investigation can be obtained solely by counts of immuno-gold stained antigens, which allow the stoichiometric detection of each specific component within its original cell compartment (Bergersen et al. 2008). So far, intense immuno-staining at light microscopy led to assume that alpha-syn represents the major component of the inclusions and the wide cytopathology developing widespread within degenerating catecholamine cells.

However, doubts remain concerning the specific protein amount just based on qualitative immuno-staining. This also leaves open a key question: how much alpha-syn compared with some other key protein does occur within inclusions? Similarly, the abundance of protein compared with non-protein components remains elusive. For instance, in the case of LB in PD, the recent work by Shahmoradian et al. (2019) detailed the ultrastructure of specific areas selected at light microscopy as strongly stained for alpha-syn. Within these regions, a remarkable amount of non-protein structures covering a great area was identified at TEM. Similarly, pioneer ultrastructural studies about inclusions led to stain some key proteins (Iwatsubo et al. 1996) and spoke up for the occurrence of multi-faceted tubulo-vesicular vacuoles. However, in this pioneer work, immuno-gold quantification was not carried out and inclusions were not analyzed in situ, but they were inferred following a gradient centrifugation. Thus, at present days, even in keeping with the mainstream assessing alpha-syn as the major component of inclusions within diseased catecholamine neurons, the amount of this protein was never counted. Similarly, a quantitative comparison between alpha-syn and other key proteins remains non-investigated. Again, the amount of non-protein structures being abundant in these areas remains non-defined. The analysis of proteins other than alpha-syn is emerging in recent studies showing the potential key role of p62 and poly-ubiquitin in seeding neuronal inclusions at early stages of catecholamine cell pathology and persisting later on in the disease course (Kurosawa et al. 2016; Noda et al. 2022; Sato et al. 2018, 2020, 2021). Still, the lack of quantitative data concerning the amount of these specific proteins and the area covered by vesicular bodies distinct from protein aggregates is in need to be investigated. Therefore, in the present study, we counted stoichiometrically specific key proteins using immuno-gold transmission electron microscopy (TEM) within alpha-syn-rich pathological compartments. These areas correspond to those identified following immuno-staining at light microscopy in METH-treated catecholamine cells. We detailed the quantitative relevance of alpha-syn compared with some key proteins such as p62 and poly-ubiquitin and we measured the protein vs. non-protein components within these areas occurring during METH-induced cytopathology.

## Materials and methods

### Experimental design

Cells were administered various doses of METH to select the optimal dose to produce the most severe alpha-syn-related pathology, while avoiding to induce extensive cell death. At this purpose, a dose–response curve showing the occurrence of cell death was compared with a dose–response curve measuring alpha-syn-related cytopathology. The chance to analyze a highly reproducible in vitro system allows to count specific protein amount within alpha-syn positive areas. The ultrastructural analysis of these alpha-syn positive cell domains following METH was developed by combining light and electron microscopy (CLEM), similarly to that recently reported by Shahmoradian et al. (2019). A preliminary approach consisted of selecting specific areas visualized at light microscopy through semi-thin sections to further proceed with electron microscopy to carry out a quantitative assessment. Semi-thin slices stained with toluidine blue provided a further analytical step to confirm the placement of those pale cytosolic areas observed at Hematoxylin & Eosin (H&E) light microscopy, which characterize METH-induced cytopathology, and corresponding to strong alpha-syn staining as confirmed by pre-embedding immuno-peroxidase. Once these areas were identified through semi-thin sections, they were further dissected to carry on ultra-thin slices for electron microscopy, where the amounts of specific antigens could be detected by immuno-gold stoichiometry. These pale eosinophilic areas represented by H&E and toluidine blue histochemistry were evidenced as encircled red-dotted line. Within these areas, which correspond to those previously immuno-stained at light microscopy, a further validation of alpha-syn content was provided by ultra-thin sections (70–90 nm) from samples undergone pre-embedding immuno-peroxidase before proceeding with the counts of immuno-gold stoichiometry of specific protein content and plain ultrastructural morphometry of membranous organelles.

Thus, the first part of the study assessed specific (alpha-syn, p62, poly-ubiquitin, ubiquitin) protein amount at light microscopy (immuno-peroxidase and immuno-fluorescence) following METH administration. The second part of the study combined light and electron microscopy (CLEM) to count protein stoichiometry at TEM within corresponding cytosolic areas possessing strong alpha-syn immuno-staining at light microscopy. In these cytosolic areas featuring a cytopathology reminiscent of inclusions, the amount of alpha-syn was compared with other proteins, such as p62 and poly-ubiquitin in situ. In fact, these latter proteins were recently claimed as early markers of catecholamine cytopathology (Kurosawa et al. 2016; Noda et al. 2022; Sato et al. 2018, 2020, 2021). This ultrastructural analysis indicates that, within alpha-syn abundant areas, p62 prevails instead. Therefore, we added a reversed sampling focusing also on those areas which expressed the highest content of p62. In both cases, areas were measured to calculate the membranous organelles (lysosomes, autophagosomes, and mitochondria) compared with protein content.

### Cell cultures

Pheochromocytoma PC12 cell cultures, purchased from IRCCS San Martino Institute (Genova, Italy), were kept in a wet atmosphere with 5% CO_2_ at 37 °C and were grown in RPMI 1640 medium (Sigma-Aldrich, St. Louis, MO, USA), supplemented with horse serum (HS, Sigma-Aldrich), fetal bovine serum (FBS, Sigma-Aldrich), and antibiotics (streptomycin and penicillin). Experiments were carried out when PC12 cells were in the log-phase of growth, corresponding to 70% confluence (Qiao et al. 2001; Song et al. 1998). Before experimental treatments, cells were seeded according to the different experimental procedures and incubated for 24 h at 37° C in 5% CO_2_. In detail, for trypan blue (TB) staining, cells were seeded at a density of 10^4^ cells/well and placed within 24-well plates in 1 mL of culture medium; for light microscopy staining procedures, 5 × 10^4^ PC12 cells were seeded on poly-lysine coverslips and placed in 24-well plates in a final volume of 1 mL/well. For TEM, 1 × 10^6^ cells were seeded in culture dishes in a final volume of 5 mL.

### Cell treatments

A stock solution of METH (kindly gifted by Forensic Medicine, University of Pisa) 10 mM was obtained by dissolving in 1 mL of culture medium 2.3 mg of METH. Aliquots of the stock solution were diluted in the culture medium to obtain the treatment solutions. In detail, PC12 cells were exposed to increasing doses of METH, ranging from 1 up to 1000 µM, for 72 h. Control cultures were kept in the same volume of culture medium for the same time interval. This time interval was selected based on previous studies (Fornai et al. 2004; Lazzeri et al. 2018, 2021). At the end of the treatments, PC12 cells were washed in PBS and processed according to the various experimental procedures. After the pilot dose–response study, the dose of METH which was selected to analyze various antigens and ultrastructural morphometry was 100 µM for 72 h.

### Hematoxylin and eosin (H&E) histochemistry

Cells were fixed in a 4% paraformaldehyde phosphate-buffered solution (PBS) for 15 min, washed in PBS, and then immersed for 15 min in the hematoxylin solution (Sigma-Aldrich). The hematoxylin staining was stopped through repeated washing in running water. After, cells were immersed within the eosin solution (Sigma-Aldrich) for a few minutes and washed out again to remove the excess of dye. Finally, after dehydration in increasing alcohol solutions, cells were clarified in xylene, covered with DPX mounting medium (Sigma-Aldrich) and observed under a Nikon Eclipse 80i light microscope (Nikon, Tokyo, Japan) equipped with a digital camera connected to the NIS Elements software for image analysis (Nikon, Tokyo, Japan).

Cell count was carried out under light microscope, using a 20 × magnification; the number of H&E-stained cells in each experimental group was counted and expressed as the mean percentage ± SEM of the control group (which corresponds to 100%). Data refer to three independent experiments.

Adobe Photoshop CS4 Extended program (version 11.0, Adobe Systems Inc., San Jose, CA, USA) was used to create the artwork.

### Fluoro-Jade B (FJB) histo-fluorescence

Cells were fixed with a solution of paraformaldehyde 4% for 5 min, washed in PBS, and immediately incubated with 0.06% potassium permanganate for 10 min at room temperature. After washing in distilled water, cells were incubated for 20 min in a FJB solution prepared by dissolving 0.01% FJB (Merck Millipore, Billerica, MA, USA) in acetic acid. Cells were incubated with 0.0004% of this FJB solution for 20 min and then cover-slipped with mounting medium. FJB-positive cells were analyzed using a Nikon Eclipse 80i light microscope (Nikon, Tokyo, Japan), equipped with a florescence lamp and a digital camera connected to the NIS Elements software for image analysis (Nikon, Tokyo, Japan).

Cell count was carried out under fluorescence microscope at 20 × magnification. The number of FJB-fluorescent cells was expressed as the mean number ± SEM for each experimental group. The intensity of the fluorescent signal was measured under florescence microscopy using the software Image J (NIH, Version 1.8.0_172, Bethesda, MD, USA) and values are expressed as the mean percentage ± SEM of optical density (assuming controls as 100%) from *N* = 90 cells/group. Data refer to three independent experiments.

Adobe Photoshop CS4 Extended program (version 11.0, Adobe Systems Inc., San Jose, CA, USA) was used to create the artwork.

### Trypan blue (TB)

Cells were collected and centrifuged at 800×*g* for 5 min, and the cell pellet was suspended in 0.5 mL of culture medium. Twenty-five µL of cell suspension was incubated for 10 min in a solution containing 1% TB in PBS and 10 µL of this solution were injected into a Bürker chamber and analyzed under an Olympus CKX 41 inverted microscope (Olympus Corporation, Tokyo, Japan). Viable and nonviable cells were counted and values were expressed as the mean percentage ± SEM of TB-positive cells out of the total cells. Data refer to three independent experiments.

### Immuno-histochemistry

After washing in PBS, PC12 cells were fixed with 4% paraformaldehyde in PBS for 15 min, and incubated with 0.1% TritonX-100 (Sigma-Aldrich) for 15 min in PBS.

For immuno-fluorescence experiments, cells were immersed for 1 h in a blocking solution containing 10% normal goat serum (NGS) in PBS at room temperature and then were incubated overnight at 4 °C in a solution containing the primary antibodies in PBS and 1% normal goat serum. In detail, the following primary antibodies (AbI) were used: (1) anti alpha-syn AbI (Abcam, Cambridge, UK), diluted 1:100; (2) anti-p62 AbI (Abcam), diluted 1:100; (3) anti-poly-ubiquitin AbI (Abcam), diluted 1:100; (4) anti-ubiquitin AbI (Sigma-Aldrich), diluted 1:100.

After rinsing in PBS, cells were incubated for 1 h with the appropriate fluorophore-conjugated secondary antibodies (i.e., Alexa 488, Life Technologies Carlsabad, CA, USA, or Alexa 594, Life Technologies) diluted 1:200. All these reactions were carried out within the well plate. After washing in PBS, cells were transferred on coverslip, mounted with the mounting medium Fluoroshield (Sigma-Aldrich), and finally observed under the Nikon Eclipse 80i light microscope (Nikon) equipped with a fluorescent lamp and a digital camera connected to the NIS Elements Software for image analysis (Nikon). Negative control cells were incubated with secondary antibodies only. For double fluorescence pictures, single fluorescent images were acquired independently, and then, they were merged using the NIS Elements Software (Nikon).

For immuno-peroxidase experiments, cells were incubated in 3% hydrogen peroxide (H_2_O_2_) for 20 min at room temperature to block endogenous peroxidase activity, and then were plunged in a blocking solution containing 10% NGS in PBS for 1 h at room temperature. Cells were incubated overnight at 4 °C with the primary antibody solution containing 2% NGS in PBS and the following AbI: the anti-alpha-syn AbI (Abcam) (1:2000), the anti-p62 AbI (Abcam, 1:2000), the anti-poly-ubiquitin AbI (Abcam, 1:2000), and the anti-ubiquitin AbI (Sigma-Aldrich, 1:2000).

The antigen–antibody reaction was revealed using the appropriate biotin-conjugated secondary antibodies (Vector Laboratories, Burlingame, CA, USA) diluted 1:200 for 1 h at room temperature, followed by avidin–biotin complex (Vector) for 1 h and the peroxidase substrate diaminobenzidine (DAB, Vector) for a few minutes. Finally, cells were dehydrated using increasing alcohol solutions. All these reactions were carried out within the well plate. After washing in PBS, cells were clarified in xylene and transferred on coverslips where DPX mounting medium (Sigma-Aldrich) was added before their observations at light microscopy (Nikon) equipped with a digital camera connected to the NIS Elements software for image analysis (Nikon, Tokyo, Japan). Negative control cells were incubated with secondary antibodies only.

The optical density of each single fluorescent or peroxidase-stained picture was measured using Image J software (NIH, Version 1.8.0_172, Bethesda, MD, USA). Values are given as the mean percentage ± SEM from *N* = 90 cells/group.

In double fluorescent pictures, merging areas were measured in µm^2^ using Image J software (NIH, Version 1.8.0_172, Bethesda, MD, USA) and values are given as the mean merging areas ± SEM per cell from *N* = 90 cells/group.

All data refer to three independent experiments.

Adobe Photoshop CS4 Extended program (version 11.0, Adobe Systems Inc., San Jose, CA, USA) was used to create the artwork.

### Sample preparation for semi-thin slices and combined light and electron microscopy (CLEM)

At the end of the treatment, cells were centrifuged at 1000×*g* for 5 min, rinsed in PBS, and fixed in a solution of 2.0% paraformaldehyde and 0.1% glutaraldehyde in 0.1 M PBS (pH 7.4) for 90 min at 4 °C. Cells were then washed out in PBS (0.1 M), post-fixed in 1% osmium tetroxide (OsO4) for 1 h, at 4 °C, and dehydrated in increasing ethanol solutions. Finally, they were embedded in Epoxy resin.

Semi-thin slices (about 1 µm thick) were cut by ultra-microtome (Leica Microsystems, Leica Microsystems, Wetzlar, Germany), and they were stained with toluidine blue and observed at light microscopy (Nikon). These slices were ordered with ultra-thin slices (70–90 nm thick) to combine light and electron microscopy (CLEM) analysis of the corresponding areas. Ultra-thin slices (70–90 nm thick) were counterstained with uranyl acetate and lead citrate to be examined using a JEOL JEM SX100 transmission electron microscope (JEOL, Tokyo, Japan).

To provide internal validation, each semi-thin slice was followed by an ultra-thin slice (each including five series), which were processed for light and electron microscopy, respectively. This allowed to compare similar cytosolic areas according to a roughly 0.1 µm thickness interval between light and electron microscopy sampling. As a reference point, in these alternate slices, we used as a spatial reference the shape of vacuolated cytosolic domains, which were poorly stained by toluidine blue (light beam) and provided a poor contrast to the electron beam. The choice of selecting highly vacuolated cytosolic domains highly stained with alpha-syn was based on light microscopy data. In fact, the occurrence of clusters of alpha-syn was abundant in these cell regions. Immuno-peroxidase carried out post hoc at pre-embedding validated the occurrence of these antigens within ultra-thin slices.

### Pre-embedding for immuno-peroxidase

At TEM, alpha-syn and p62 were also labeled by immuno-peroxidase. Cell pellets were fixed with 2.5% paraformaldehyde and 0.1 glutaraldehyde dissolved in PBS for 90 min. After washing in PBS, cell pellets were incubated in 0.002% hydrogen peroxide in 0.05 M Tris–HCl buffer, pH 7.6 for 2.5 min. Then, after washing in PBS, cell pellets were permeabilized in ethanol (10% for 5 min, 25% for 5 min, and 10% for 5 min) and pre-blocked with a solution containing 10% NGS and 0.2% saponin in PBS for 30 min. Samples were then incubated with anti-alpha-syn AbI (1:100, Abcam) or anti-p62 AbI (1:100, Abcam) diluted in 10% NGS and 0.2% saponin in PBS for 24 h. Then, samples were incubated with a solution containing the biotin-conjugated secondary antibodies (Vector) diluted 1:100 for 1 h at room temperature. After washing in PBS, samples were incubated in avidin–biotin peroxidase complex (Vector) for 1 h. After washing in PBS samples were incubated in 0.075% DAB (Vector) for a few minutes.

Samples were washed in PBS and osmicated, dehydrated, and embedded in Epoxin resin. Ultra-thin sections were cut by ultra-microtome (Leica Microsystems) and were observed under TEM (JEOL JEM SX100, JEOL, Tokyo, Japan).

Adobe Photoshop CS4 Extended program (version 11.0, Adobe Systems Inc., San Jose, CA, USA) was used to create the artwork.

### Post-embedding for immuno-gold TEM

Ultra-thin slices from cell cultures were collected on nickel grids, and they were de-osmicated in aqueous solution saturated by sodium metaperiodate (NaIO4), for 15 min. Sections were washed three times for 10 min in ice-cold filtered PBS (pH 7.4) and the grids were treated with ice-cold PBS containing 10% NGS and 0.2% saponin to block non-specific antigens for 20 min at room temperature.

Primary antibodies were incubated in ice-cold PBS containing 1% NGS and 0.2% saponin in a humidified chamber overnight, at 4 °C. The following primary antibodies were used: anti-alpha-syn AbI (1:100, Abcam); anti-p62 AbI (1:100, Abcam); anti-poly-ubiquitin AbI (1:100, Abcam); anti-ubiquitin AbI (1:100, Sigma-Aldrich). Double immuno-gold staining was used to compare the amount and co-localization of the following: alpha-syn and p62, alpha-syn and poly-ubiquitin, and p62 and poly-ubiquitin.

Stoichiometry staining was obtained through a solution containing gold-conjugated secondary antibodies (gold particle diameter, 10 nm or 20 nm, BB International, Cardiff UK) diluted 1:100, in PBS containing 1% goat serum and 0.2% saponin for 1 h, at room temperature. The size of immuno-gold particles was switched to validate the non-relevance of steric encumbrance in double immuno-staining. After rinsing in PBS, grids were incubated in 1% glutaraldehyde for 3 min, and they were washed in distilled water and further stained with uranyl acetate and lead citrate. Ultra-thin sections were finally observed at TEM (JEOL JEM SX100). Control sections were incubated with secondary antibodies only.

The number of immuno-gold particles related to alpha-syn, p62 and poly-ubiquitin proteins was expressed as the mean ± SEM within 2 µm^2^ selected areas from *n* = 30 cells per group.

Area of the tubulo-vesicular membranes and the cytosol within 2 µm^2^ selected areas was measured using Image J software (NIH, Version 1.8.0_172, Bethesda, MD, USA) at 6000 ×. Values are given as the mean percentage ± SEM from *n* = 30 cells/group.

Electron density of 2 µm^2^ selected areas was measured using Image J software (NIH, Version 1.8.0_172, Bethesda, MD, USA) at 6000 ×. Values are given as the mean percentage ± SEM of electron density measured in METH-treated cells compared with electron density measured in control cells (assumed as 100%) from *n* = 30 cells/group.

Adobe Photoshop CS4 Extended program (version 11.0, Adobe Systems Inc., San Jose, CA, USA) was used to create the artwork.

### Extended statistics

In this paragraph, we report classic descriptive and inferential statistics, which were implemented by an extended explanation of arbitrary criteria used here to provide the optimal sampling for each procedure. A brief comment about validation of the procedure is provided as well. The effects of various doses of METH ranging from 1 to 1000 µM on cell damage (H&E) were expressed as the mean percentage of healthy cells ± SEM compared with control. The number of degenerating cells (FJB) was expressed as the mean ± SEM of stained cells. Degenerating cells visualized at TB was expressed as the percentage of stained cells within the whole population. The difference between groups were assessed by ANOVA with Sheffe’s post hoc analysis; *H*_0_ was rejected for *p* < 0.05.

The issue of combining different procedures and different techniques to count cell damage at light microscopy may produce discrepancies based on the specific procedure. For instance, the amount of severe cell damage assessed by H&E is based on the actual lack of cell structures (unique among light microscopy procedures used here) and the presence of remarkable alterations of cell shape and size and faint cytosol visible as pale eosinophilic cytosolic areas. In keeping with H&E staining, the amount of cell damage included those cells, where these alterations were severe. In the case of FJB, the count is based on the fluorescent area conventionally assuming that FJB immuno-fluorescence is synonymous of dying cell (which still may not be constantly true considering our lack of an in-depth knowledge of which and how many markers are responsible for FJB-induced fluorescence). In this case, additional bias may lead to non-damage dependent occurrence of some molecules, which target FJB, thereby providing a bias. This may explain why the amount of cell death assessed using FJB was the highest compared with all other methods (still considering that this may also depend on the highest sensitivity of this procedure). Data about cell death counted at TEM were inferred by counting the decrease of viable cells compared with control. Despite slight differences, the consistency across different techniques and between light and electron microscopy was remarkable concerning the amount of METH-induced cell damage, and values were quite steady, which internally validate each single procedure applied here.

Data about immuno-fluorescence for alpha-syn, p62, poly-ubiquitin, and ubiquitin stained alone or in combination (merging) were expressed as semi-quantitative fluorescent densitometry considering the fluorescent area of merging comparing controls with METH 100 µm. The comparison was carried out using ANOVA with Sheffe’s post hoc analysis; *H*_0_ was rejected for *p* < 0.05. This rough calculation due to intrinsic limits of non-linear relationship between protein amount and immuno-staining serves as a guide to better address molecular quantification of these proteins at TEM. Alpha-syn positive areas were selected by counting immuno-gold particles within selected circular areas of 2 µm^2^. This size was selected based on sampling the distribution of immuno-gold clusters observed in alpha-syn hot spots at CLEM in METH-treated cells. These consistently overlap with pale eosinophilic areas. The amount of immuno-gold for alpha-syn within these areas was different within various cytosolic domain ranging from 0 up to 12 immuno-gold. An arbitrary cut-off was set at 7 immuno-gold alpha-syn particles which was arbitrarily selected to define at sub-cellular level a cluster of alpha-syn. Within these areas of high alpha-syn content, the amount of p62 was counted as well. In this case, we used different immuno-gold particles owing different diameter (either 10 nM or 20 nM, respectively) to distinguish both antigens in the same ultra-thin section. In parallel experiments, we assessed that the diameter of the immuno-gold particles did not affect the count of the antigen, likely due to a lack of allosteric interference between the immuno-gold particles and primary antibodies on antigen epitopes. The comparison between p62 and alpha-syn immuno-gold was carried out using ANOVA with Sheffè’s post hoc analysis. Since p62 prevails at large compared with alpha-syn even in those areas being selected as the richest in alpha-syn content, a second step was necessary to re-assess the relative amount of these proteins by starting to select areas where p62 was most abundant (cut-off 100 immuno-gold particles per 2 µm^2^), independently by the amount of alpha-syn. Despite some overlapping between these antigens occurs, we found that a different placement was evident at electron microscopy, which did not emerge when the localization of both antigens was detected at light microscopy. In fact, the richest p62 immuno-gold areas analyzed at TEM may not contain high alpha-syn. Again, at TEM compared with light microscopy, the occurrence of p62 was strikingly more abundant than alpha-syn. Such a difference was magnified when alpha-syn and p62 richest areas were calculated following METH administration. In these experimental conditions, these 2 µm^2^-wide areas were analyzed concerning their structure. In fact, within these areas, the amount of altered lipid membranous organelles was counted as well. In detail, the area taken by membrane limited organelles (autophagosomes, lysosomes and mitochondria) was calculated independently within alpha-syn and p62 rich areas. Despite some areas feature a high content of both antigens, we found that vesicles limited organelles were more abundant within areas identified by p62 immuno-gold staining compared with alpha-syn immuno-gold staining. Within these areas, the mean electron-density was calculated. In calculating the specific kind of membranous organelles, we purposefully did not discriminate between the amounts of mitochondria compared with lysosomes or autophagosomes to harvest all membranous structures as previously described (Shahmoradian et al. 2019). The nuclear area was never considered in keeping with the cytosolic nature of the pathological process under analysis. In selecting the placement, the counted area was placed around the protein cluster under primary analysis. This led to discrepant, only partially overlapping regions depending on which protein was primarily counted. Again, a discrepancy exists considering non-protein vesicle crowding. This partial overlapping indicates that the region shape and size varies depending on which structure is considered as the hallmark. The choice of the protein hallmark was done at first based on classic literature (alpha-syn), and then, it was slightly modified based on actual quantitative findings (the excess of p62). Concerning the pattern of p62, we noticed a remarkable packing where p62 and poly-ubiquitin were expressed densely. The size of these small circular areas providing a sort of p62/poly-ubiquitin domain was roughly tenfold lower compared with the circles selected for alpha-syn clusters. Therefore, a further count was used within these 200 nm^2^ areas to better express the density of p62 and to make a comparison with the density of alpha-syn.

From a statistical perspective and a methodological approach, we found strong discrepancies between immuno-fluorescence/immuno-peroxidase at light microscopy and immuno-gold at TEM. In detail, at light microscopy, most cells appear to stain for alpha-syn and p62 according to an all (METH) or none (control) pattern. This contrasts with TEM showing a twofold difference between controls and METH. This is true also concerning various cell domains. This suggests that the statistical power of TEM to paint a scenario which is adherent to actual cytopathology is enormous compared with light microscopy. This latter procedure appears to provide negligible staining up to a level where a sort of dramatic staining enhancement takes place.

The striking discrepancy between immuno-stained area observed at light microscopy (almost as a clear-cut region compared with surrounding cytosol) with the faint, undefined border under TEM is due to the striking difference in magnification, which either neglects or highlights the continuum of cytopathology leading to undefined borders for these multi-faceted biological structures observed at high magnification.

## Results

### METH induces dose-dependent cell damage

**Fig. 1:**
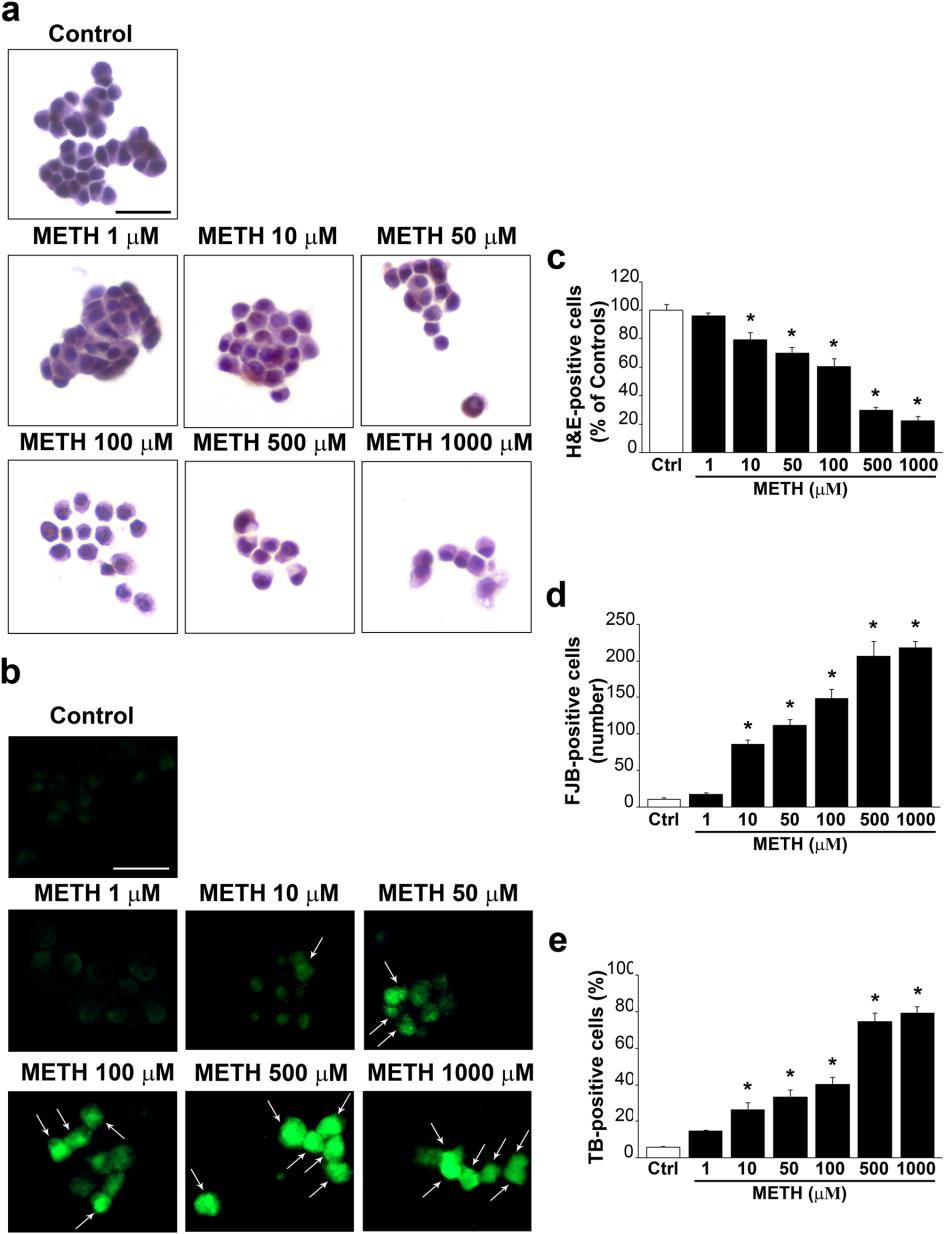
METH dose-dependently increases cell damage. Representative **a** H&E- and **b** FJB-stained pictures from controls and following increasing doses of METH (1 µM, 10 µM, 50 µM, 100 µM, 500 µM, 1000 µM). Arrows indicate intensely FJB-positive cells. The graphs report: **c** the count of viable H&E-positive cells, which quantifies cell survival, expressed as percentage of controls; **d** the number of FJB-positive cells, which quantifies degenerating cells; **e** the percentage of TB-positive cells, which quantifies dying cells. *Ctrl* controls. Scale bars: 30 µm (**a**, **b**). *p < 0.05 compared with controls

**Fig. 2:**
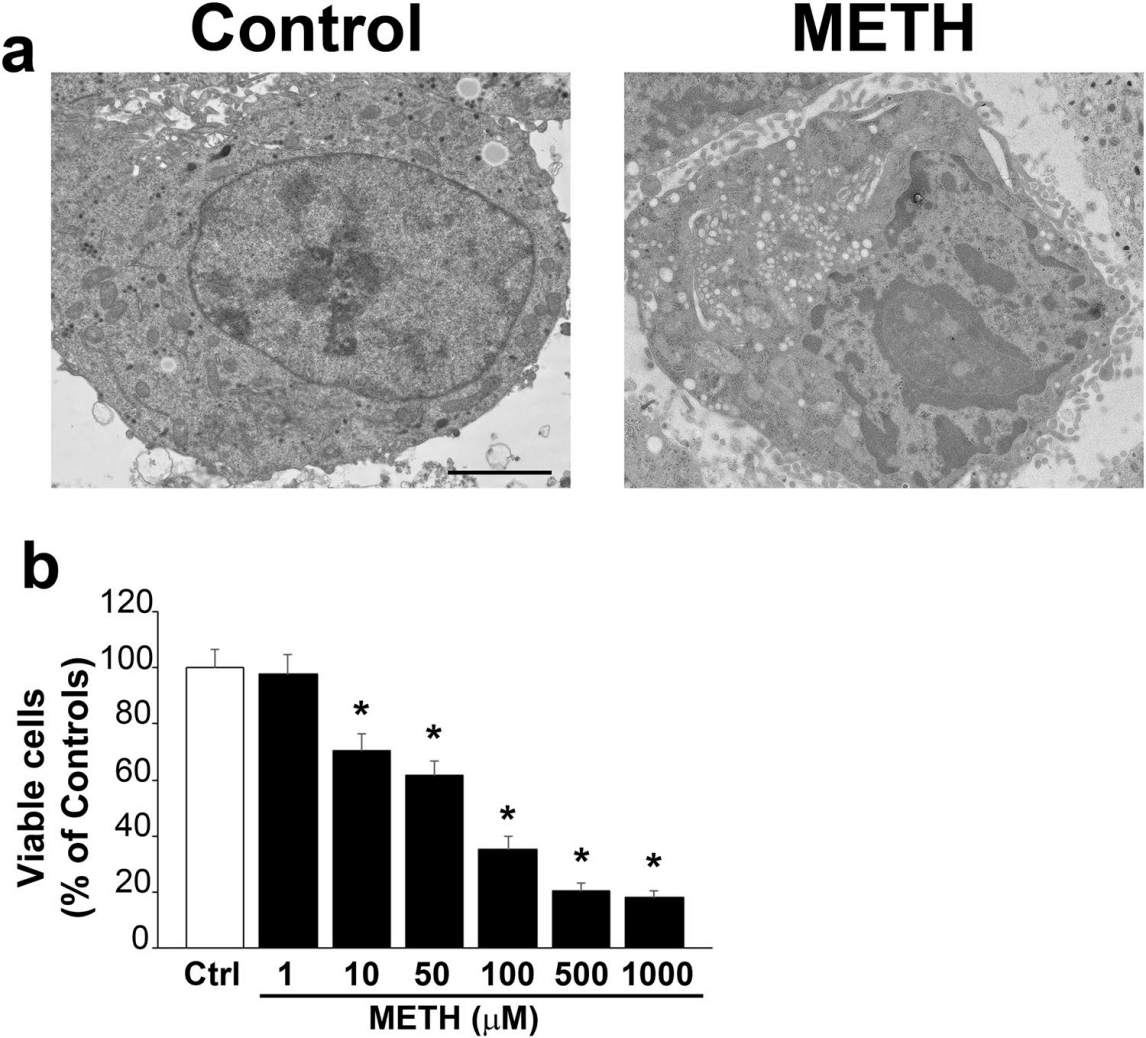
METH dose-dependently decreases viable cells counted at TEM. **a** Representative pictures at TEM from a control healthy cell and a damaged cell exposed to METH 100 µM. The occurrence of cell damage was assessed, as reported in the text, independently by the ongoing cell pathology (including both apoptosis and necrosis as well as non-specific severely altered cells). **b** The graph reports the counts of viable cells following increasing doses of METH (1 µM, 10 µM, 50 µM, 100 µM, 500 µM, 1000 µM), which are expressed as percentage of controls. *Ctrl* controls. Scale bar: 1.6 µm. *p < 0.05 compared with controls

As reported from light microscopy of Fig. 1 increasing doses of METH ranging from 1 up to 1000 µM increase cell damage. Figure 1a reports representative pictures from controls and following METH 1 µM, 10 µM, 50 µM, 100 µM, 500 µM, 1000 µM (H&E histochemistry). Similar representative images are reported in Fig. 1b following FJB staining, since the fluorescent dye stains degenerating neurons. These effects were quantified in the graph of Fig. 1c reporting the percentage of cell damage compared with control (H&E), and in the graph of Fig. 1d, the number of degenerating cells (FJB-positive cells) is reported, while the graph of Fig. 1e reports the percentage of dying cells (TB-positive cells). These effects were confirmed by representative pictures of Fig. 2a showing a control cell and a cell exposed to METH 100 µM. In the graph of Fig. 2b, a dose–response effect following the same doses of METH reported in the graphs of Fig. 1c–e shows progressive cytopathology induced by METH at electron microscopy. In fact, Fig. 2a reports a representative control cell and a degenerating cell under the effects of METH, 100 µM. When the count of cell death was carried out at TEM, the occurrence of viable cells compared with control was considered. This means that dying cells (as the one shown in representative Fig. 2a) were not considered as part of viable cells (as extensively reported in the statistics concerning the sampling procedures). This criterion allows to consider severely damaged cells along with cell remnants, cell loss (considering both cell apoptosis and cell necrosis which occur concomitantly). Such a criterion replicates at higher resolution and finer detection what carried out during counts of H&E-stained cells, where severe cell damage was ruled out from the count of healthy, viable cells. The power of electron microscopy provides the finest evaluation and detection about severity of cell alterations. It is remarkable that, independently by the procedure used at light microscopy, the amount of cell damage produced by METH is comparable. This leads to roughly 80% of cell damage following the highest METH doses. The procedure which seems to provide the highest sensitivity to detect METH-induced cell damage is FJB which stains almost the total cell population (95.24% compared with 4.76% of controls). In these conditions, H&E and TB provide a percentage of damage slightly exceeding 70%, while TEM measured a cell damage which barely exceeds 80%. These slight discrepancies may be due to either variations in experimental conditions or the different sensitivity between techniques being used. Nonetheless, the consistency across different techniques was remarkable and it was still evident comparing light with electron microscopy. For a detailed comment concerning data variability based on each procedure, please refer to Paragraph of Supplementary Information (file 1).

### METH induces dose-dependent alpha-syn immuno-staining

**Fig. 3:**
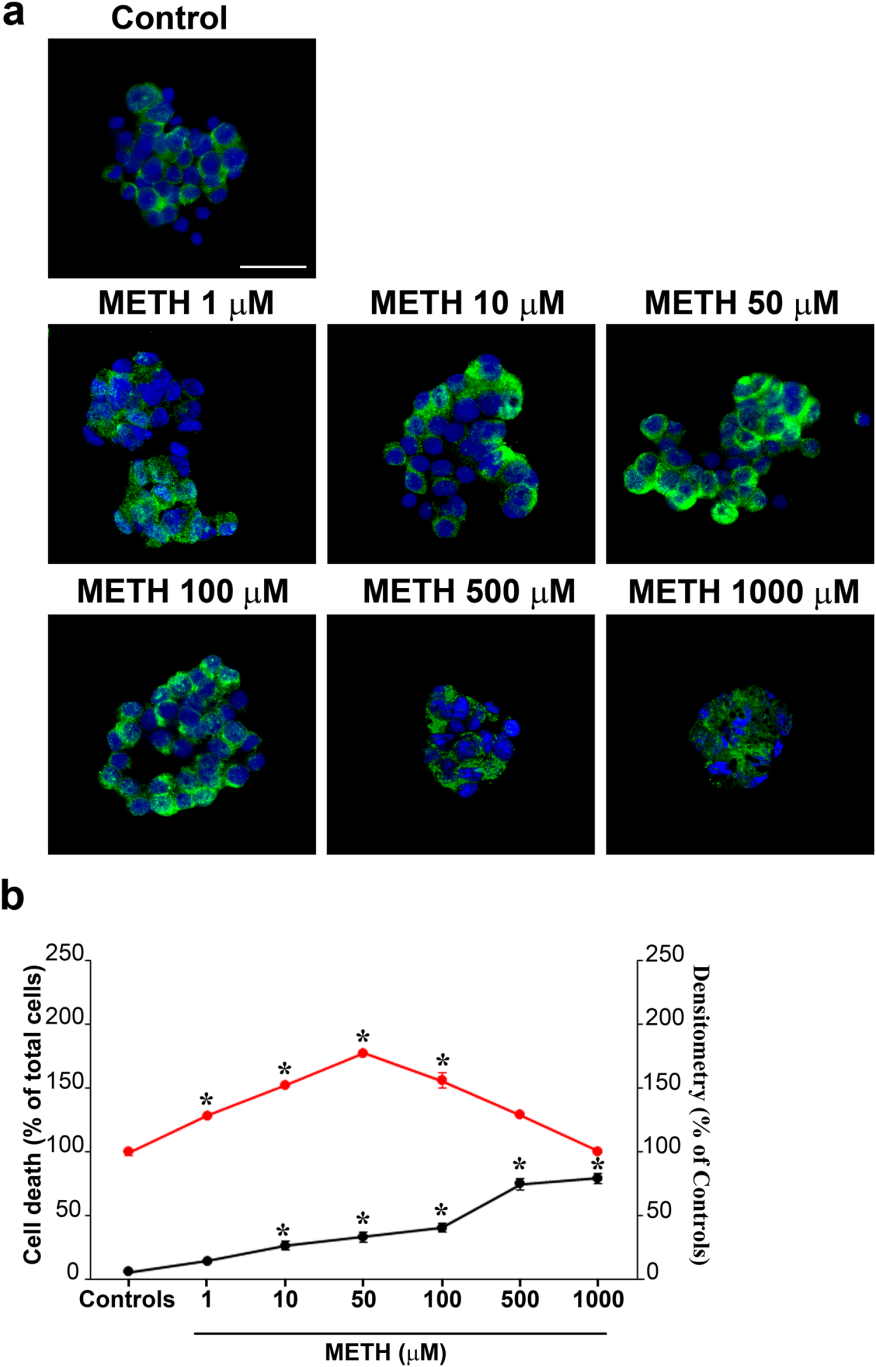
METH dose-dependently increases alpha-syn immuno-staining*.* **a** Representative pictures showing alpha-syn immuno-fluorescence in controls and following increasing doses of METH from 1 up to 1000 µM. **b** The graph reports the semi-quantitative densitometry of alpha-syn immuno-fluorescence (red dots), which progressively increases up to 100 µM and decreases for the highest doses (500 µM and 1000 µM). This dose–response curve is shifted to the left compared with that reporting METH-induced cell death (black dots). Scale bar: 30 µm. *p < 0.05 compared with controls

As reported in representative Fig. 3a, increasing the doses of METH from 1 up to 1000 µM generates a progressive increase in alpha-syn immuno-staining up to 100 µM which decreases for the highest doses. This dose–response curve for the occurrence of alpha-syn immuno-staining following METH is shifted to the left compared with the one of METH-induced cell death (graph of Fig. 3b), which indicates that an increase in alpha-syn does not overlap with frank toxicity and it is as a slighter effect compared with frank toxicity induced by high doses of METH. In fact, the high doses of METH do not produce a noticeable increase in alpha-syn, which is reduced when cell alterations are abundant.

### METH-induced increase in alpha-syn is concomitant with p62, poly-ubiquitin, and ubiquitin immuno-staining

**Fig. 4:**
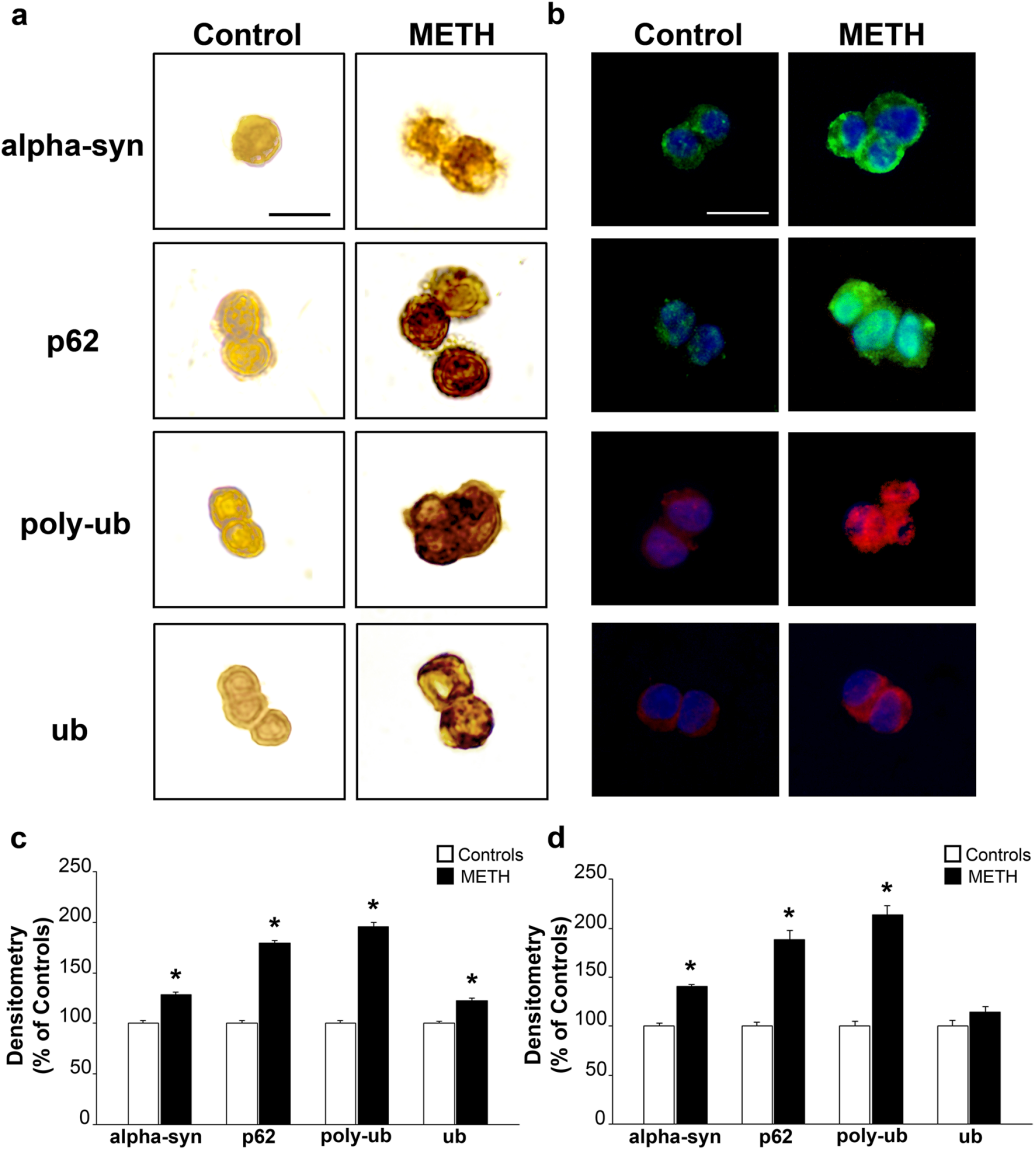
Immuno-staining for alpha-syn, p62, poly-ubiquitin, and ubiquitin following METH (100 µM) administration. Representative pictures of **a** single immuno-peroxidase and **b** single immuno-fluorescence for alpha-syn, p62, poly-ubiquitin, and ubiquitin following vehicle or METH (100 µM) administration. Semi-quantitative densitometry of immuno-peroxidase and immuno-fluorescence for each antigen is reported in the graphs **c** and **d**, respectively. Poly-ub: poly-ubiquitin; ub: ubiquitin. The effects of METH administration are remarkable in increasing the amount of immuno-peroxidase and immuno-fluorescence for alpha-syn, p62, and poly-ubiquitin, while the effects on ubiquitin are negligible. Scale bars: 30 µm (**a**, **b**). *p < 0.05 compared with controls.

Since the dose–response for METH-induced increase in alpha-syn indicates that a dose of METH 100 µM is enough to produce a plateau following semi-quantitative densitometric measurement of the protein, we selected such a dose to compare other proteins, which are involved in cell clearance and are abundant in the cytopathology of catecholamine neurons, such as p62, poly-ubiquitin, and ubiquitin (representative pictures of Fig. 4a). In this part of the study, single immuno-peroxidase implemented the immuno-fluorescence for each specific antigen (representative pictures of Fig. 4a and b, respectively).

**Fig. 5:**
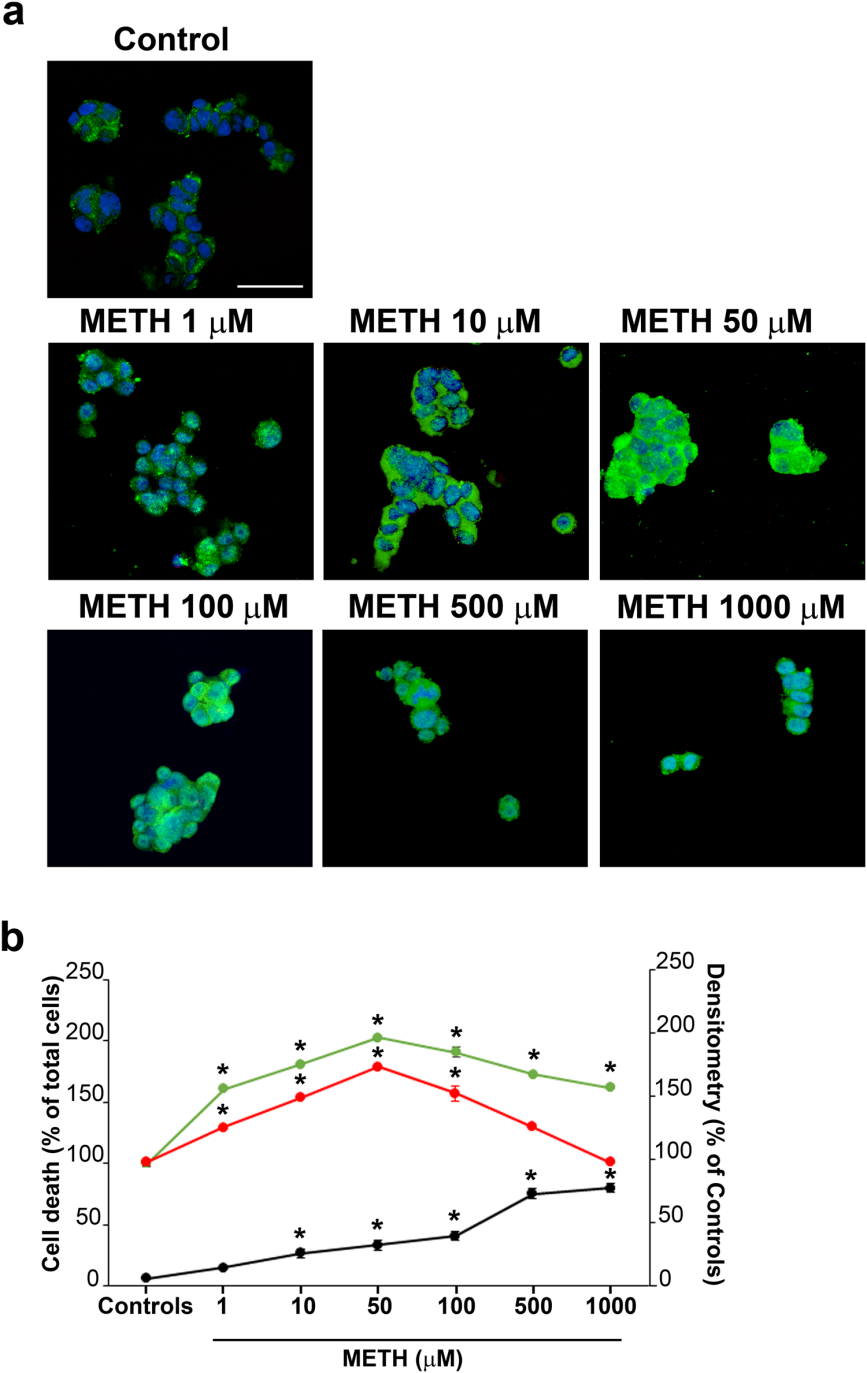
Dose–response increase of METH-induced p62 immuno-fluorescence*.* **a** Representative pictures showing p62 immuno-fluorescence in controls and following increasing doses of METH (1 µM, 10 µM, 50 µM, 100 µM, 500 µM, 1000 µM). **b** The graph reports the cumulative semi-quantitative counts of p62 densitometry (green dots), which is similar to alpha-syn densitometry (red dots). Both dose–response curves for p62 and alpha-syn are shifted to left compared with the dose-dependent increase of METH-induced cell death (black dots). In fact, the highest doses of METH (500 µM and 1000 µM) induce massive cell damage, which occludes antigen detection. Scale bar: 30 µm. *p < 0.05 compared with controls

In these experimental conditions, the semi-quantitative densitometry at immuno-peroxidase (graph of Fig. 4c) and immuno-fluorescence (graph of Fig. 4d) roughly indicates that proteins other than alpha-syn were robustly increased by METH. This was the case of p62 and poly-ubiquitin. To firmly assess such a statement, a further dose–response curve was replicated for p62 as reported in representative Fig. 5a and graph of Fig. 5b, which confirms an increase of this antigen following METH, which surpasses the increase observed for alpha-syn. As reported in the graph of Fig. 5b, the dose–response curve for p62 was analogous to the one obtained for alpha-syn and dose-dependent increase of both antigens was shifted to left compared with increase in frank cell toxicity.

In all cases, the dose of METH, 100 µM was able to induce the maximal increase of both alpha-syn and p62, still considering the increase of p62 as more relevant than alpha-syn for most doses of METH. Similar to alpha-syn, the highest doses of METH reduced the amount of p62, which is likely to depend on a severe impairment of protein integrity and protein synthesis in the presence of massive cell toxicity.

### METH induces variable merging between proteins

**Fig. 6:**
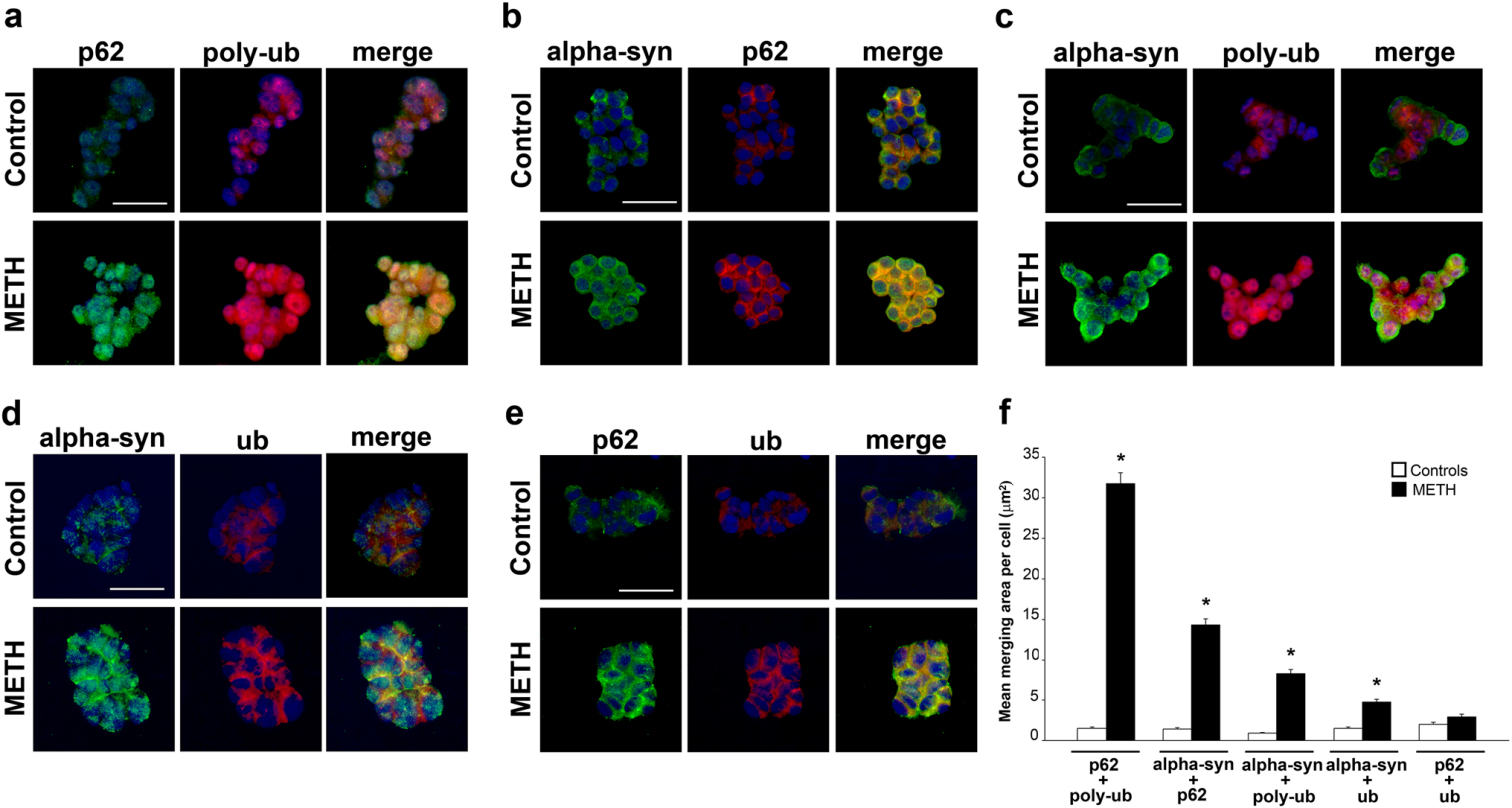
Merging of immuno-fluorescence for alpha-syn, p62, poly-ubiquitin, and ubiquitin following METH (100 µM) administration. Representative pictures show single and merging immuno-fluorescence for p62 and poly-ubiquitin (**a**), alpha-syn and p62 (**b**), alpha-syn and poly-ubiquitin (**c**), alpha-syn and ubiquitin (**d**), and p62 and ubiquitin (**e**) in control cells and cells administered METH (100 µM). **f** The graph reports the mean area of merging per cell for each antigen as indicated by each couple of bars (in each couple, white bars indicate controls and black bars indicate METH, 100 µM). The data are ordered according to decreasing immuno-fluorescence, where p62 + poly-ubiquitin produce the highest merging, while p62 + ubiquitin produces the lowest merging, where control cells and METH (100 µM)-treated cells did not differ significantly. Poly-ub: poly-ubiquitin; ub: ubiquitin. Scale bars: 32 µm (**a**, **b**); 27 µm (**c**, **e**); 18 µm (**d**). *p < 0.05 compared with controls

As shown in Fig. 6, a number of merging at immuno-fluorescence was carried out between alpha-syn, p62, poly-ubiquitin, and ubiquitin to get all the potential combinations. These experiments were designed to roughly assess the overlapping of METH-induced increase of these antigens. Representative pictures (Fig. 6a–e) and bars in graph of Fig. 6f are reported in a series showing semi-quantitative amounts. Thus, in Fig. 6a, the highest merging occurs when immuno-fluorescence of p62 overlap with immuno-fluorescence for poly-ubiquitin (see the first couple of bars of the graph of Fig. 6f). In Fig. 6b, the remarkable merging between alpha-syn + p62 is reported, which is still considerably lower compared with p62 + poly-ubiquitin (see representative pictures and compare bars of graph of Fig. 6f). A further decrease in merging was evident for alpha-syn + poly-ubiquitin in representative Fig. 6c. The merging was negligible when ubiquitin was used instead of poly-ubiquitin either in combination with alpha-syn (representative Fig. 6d) or p62 (Fig. 6e). The paucity of such a merging is evident from the last couples of bars of Fig. 6f.

These data at light microscopy suggest a number of points: (1) p62 and poly-ubiquitin are mostly induced during slight METH toxicity within catecholamine neurons; (2) cell areas where the increase of p62 and poly-ubiquitin occurs are overlapping; (3) A close spatial relationship needs to be present between p62 and poly-ubiquitin molecules; (4) the increased merging between p62 and poly-ubiquitin surpasses at large the merging between alpha-syn and poly-ubiquitin; (5) although the merging between p62 and alpha-syn is remarkable, p62 appears to increase much more compared with alpha-syn and its placement is rather closer to poly-ubiquitin than alpha-syn. All these points are key in degenerating catecholamine cells and require a solid quantitative approach to be validated. Therefore, the study proceeded through immuno-gold based protein stoichiometry to ultimately assess these findings at TEM. To work at stoichiometry in comparable cell domains where light microscopy was carried out, specimen for TEM was selected from specimen at light microscopy.

### Combined light and electron microscopy (CLEM)

#### Combined alpha-syn and p62 stoichiometry within light microscopy tracked alpha-syn positive spots

**Fig. 7:**
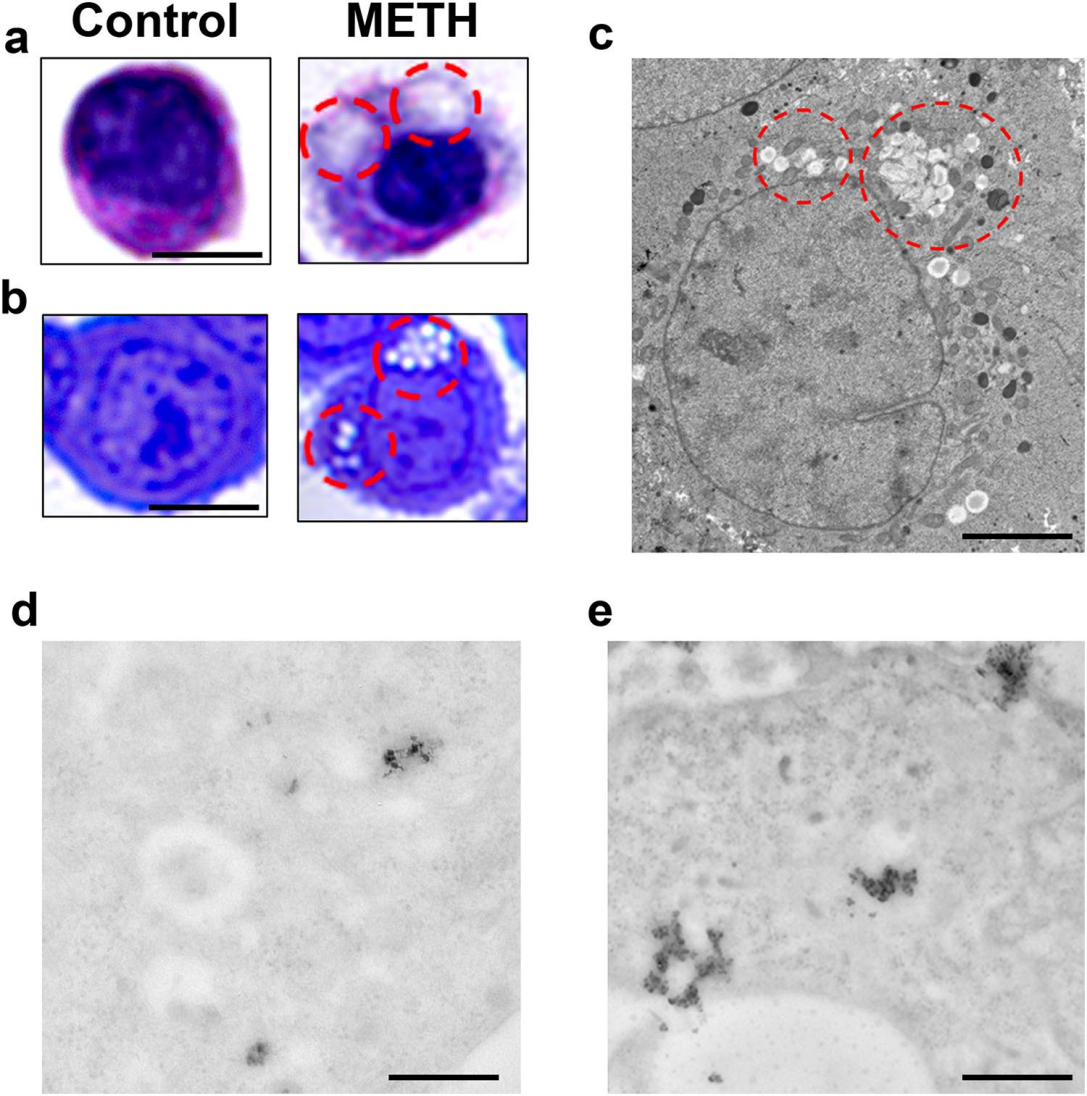
Combined light and electron microscopy (CLEM) following METH (100 µM). **a** Representative pictures at light microscopy from a control cell and a cell treated with METH (100 µM). Pictures show the following: **a** H&E histochemistry carried out on 10 µm-thick slices; **b** toluidine blue histochemistry carried out on semi-thin slices (1 µm-thick). Pathological pale cytosolic regions (red-dotted circles) are evident following METH in H&E-stained cell (**a**); these correspond to pale areas (red-dotted circles) appearing following METH in the cell stained by toluidine blue (**b**). These pale regions at light microscopy were analyzed concerning ultrastructure at TEM within ultra-thin slices (70–90 nm-thick) from METH-treated cell. This shows a number of vesicular structures (red-dotted circles), mostly placed in peri-nuclear position (**c**). Representative pictures after pre-embedding at TEM confirm that these areas are stained by immuno-peroxidase revealing primary antibodies against alpha-syn (**d**) and p62 (**e**). Scale bars: 4.5 µm (**a**, **b**); 1.7 µm (**c**); 2.3 µm (**d**, **e**)

**Fig. 8:**
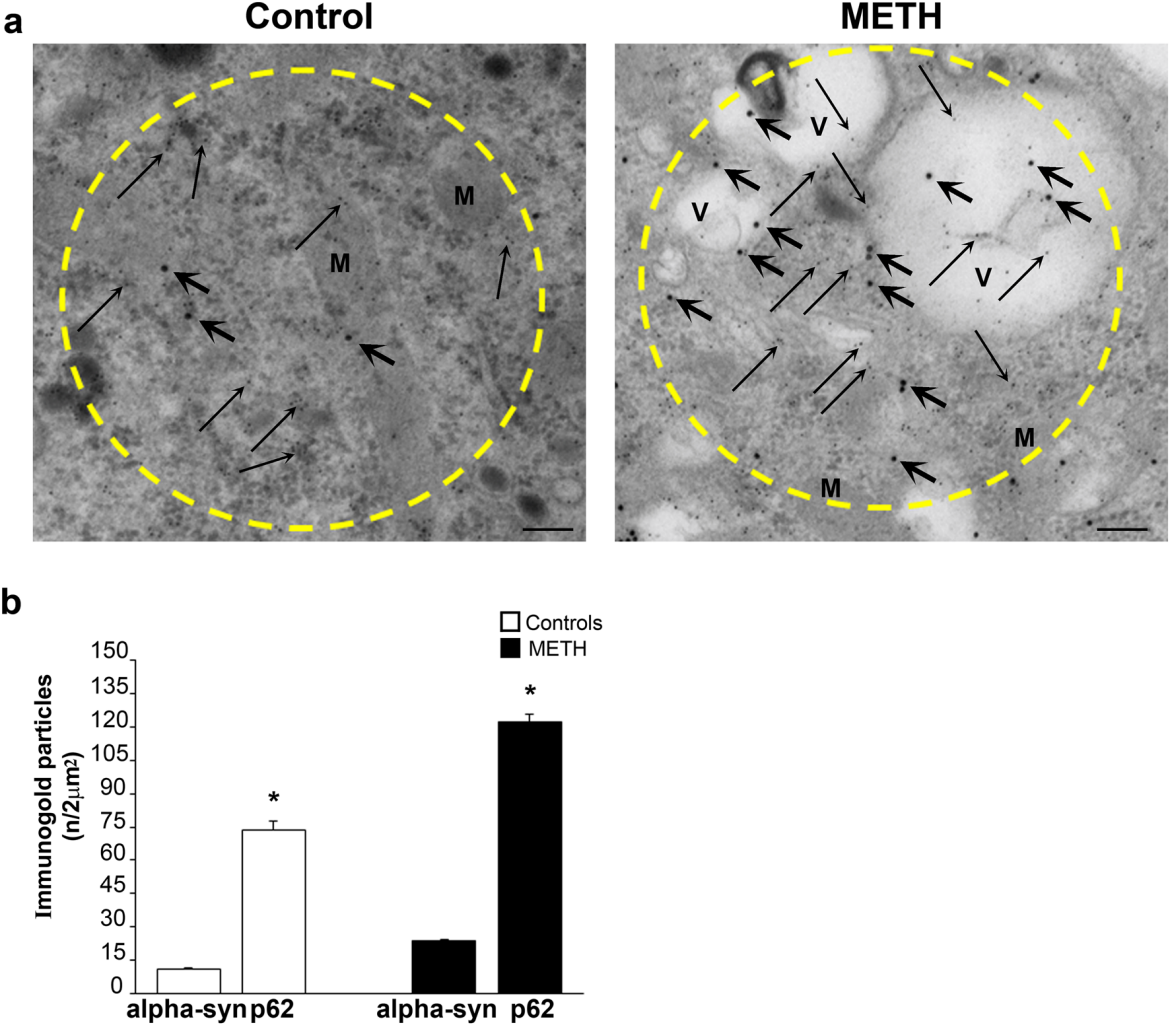
Stoichiometric counts of alpha-syn and p62 proteins bound to immuno-gold particles within alpha-syn-rich regions. **a** Representative immuno-electron microscopy pictures show specific cytosolic regions selected for the highest alpha-syn content. These regions were selected owing a diameter of 2 µm^2^. This size is indicated in the pictures by yellow dotted circles and it was chosen following a sampling from controls and METH (100 µM)-treated cells (for details, see the paragraph Extended Statistics within the Methods section). Within these areas being selected based on high alpha-syn content, double immuno-gold is carried out against alpha-syn (20 nm immuno-gold particles, shown by thick arrows) and against p62 (10 nm immuno-gold particles, shown by thin arrows). It is evident the rich amount in vesicular structures within these 2 µm^2^-wide regions, which occurs within METH-treated cells. **b** The graph reports the number of immuno-gold particles for alpha-syn and p62 counted within these 2 µm^2^ areas. It is evident that, despite these regions were selected based on high alpha-syn content, the amount of p62 prevails at large. Such a difference is evident already in baseline conditions and it is magnified following METH. *M* mitochondria, *V* vesicle. Scale bar: 180 nm. *p < 0.05 compared with controls.

To analyze at ultrastructural stoichiometry in situ the occurrence of key proteins following METH toxicity, we combined light and electron microscopy. This procedure allows a progressive increase in magnification of at least 100 ×, which has a profound impact on the morphology of inclusions and immuno-staining. Thus, one should not be surprised that classic morphology detailed at light microscopy following alpha-syn immuno-staining is unrecognizable when observed at TEM. In detail, following METH, H&E staining provided pale eosinophilic areas corresponding to immuno-stained cytosolic regions. These areas were stained either for alpha-syn or p62 at pre-embedding immuno-peroxidase. Therefore, we selected these pale areas to carry on semi-thin sections as shown in Fig. 7 as an intermediate step. As reported in representative Fig. 7a, pathological pale areas following H&E at light microscopy slices (10 µm thick), correspond to pale areas following toluidine blue in semi-thin slices (1 µm thick, Fig. 7b). These pale cytosolic regions were negligible in control cells (representative pictures of Fig. 7a, b). When ultrastructural analysis was carried out within these pale regions identified by toluidine blue-stained semi-thin slices, ultra-thin slices were cut (70–90 nm thick) to observe a number of vesicular structures, which were identified mostly in peri-nuclear position as shown by dotted encircled regions of Fig. 7c. These structures were not even considered by previous studies approaching immuno-stained regions at light microscopy. This step allows to select arbitrarily pathological cell regions based on the abundance of specific proteins. In fact, when immuno-peroxidase was analyzed at TEM, these areas still maintain both alpha-syn (representative Fig. 7d) and p62 staining (representative Fig. 7e). Within these sub-cellular regions, the stoichiometric count of each key proteins, alone or in combination, was carried out. CLEM was performed to progressively magnify the spatial resolution of analysis starting from roughly immuno-stained light microscopy areas and progressing up to single proteins though TEM stoichiometry within similar cell spots. This enables to assess the quantitative amount, while comparing the occurrence of various key proteins in METH-induced pathology at stoichiometry in situ. In this process, we started to count within alpha-syn rich areas. Based on the spatial distribution of increase in alpha-syn at first, we selected arbitrarily circles owing an area of 2 µm^2^ (as specified in the Methods section in the paragraph “Extended statistics”). These areas consistently overlap with pale eosinophilic regions. The amount of immuno-gold for alpha-syn within these areas was different within various cytosolic domains ranging from 0 up to 12 immuno-gold. An arbitrary cut-off was set at 7 immuno-gold alpha-syn particles to define at sub-cellular level a cluster of alpha-syn. Within these areas, which were selected based on high alpha-syn content, the amount of p62 was counted concomitantly (immuno-gold particles of 20 nm for alpha-syn and 10 nm for p62). Remarkably, even in these areas, the amount of p62 particles were way in excess compared with alpha-syn. This was mostly evident following METH (representative Fig. 8a). When looking at these areas abundant vesicular structures are evident as previously reported. Remarkably, following METH, the amount of both alpha-syn and p62 increased; however, the comparison between these antigens indicates that p62 increases much more than alpha-syn (Fig. 8b). Therefore, we asked how much p62 could be counted when areas were selected just based on the highest amount of p62, independently of alpha-syn.

#### Combined alpha-syn and p62 stoichiometry within p62 tracked positive spots

**Fig. 9:**
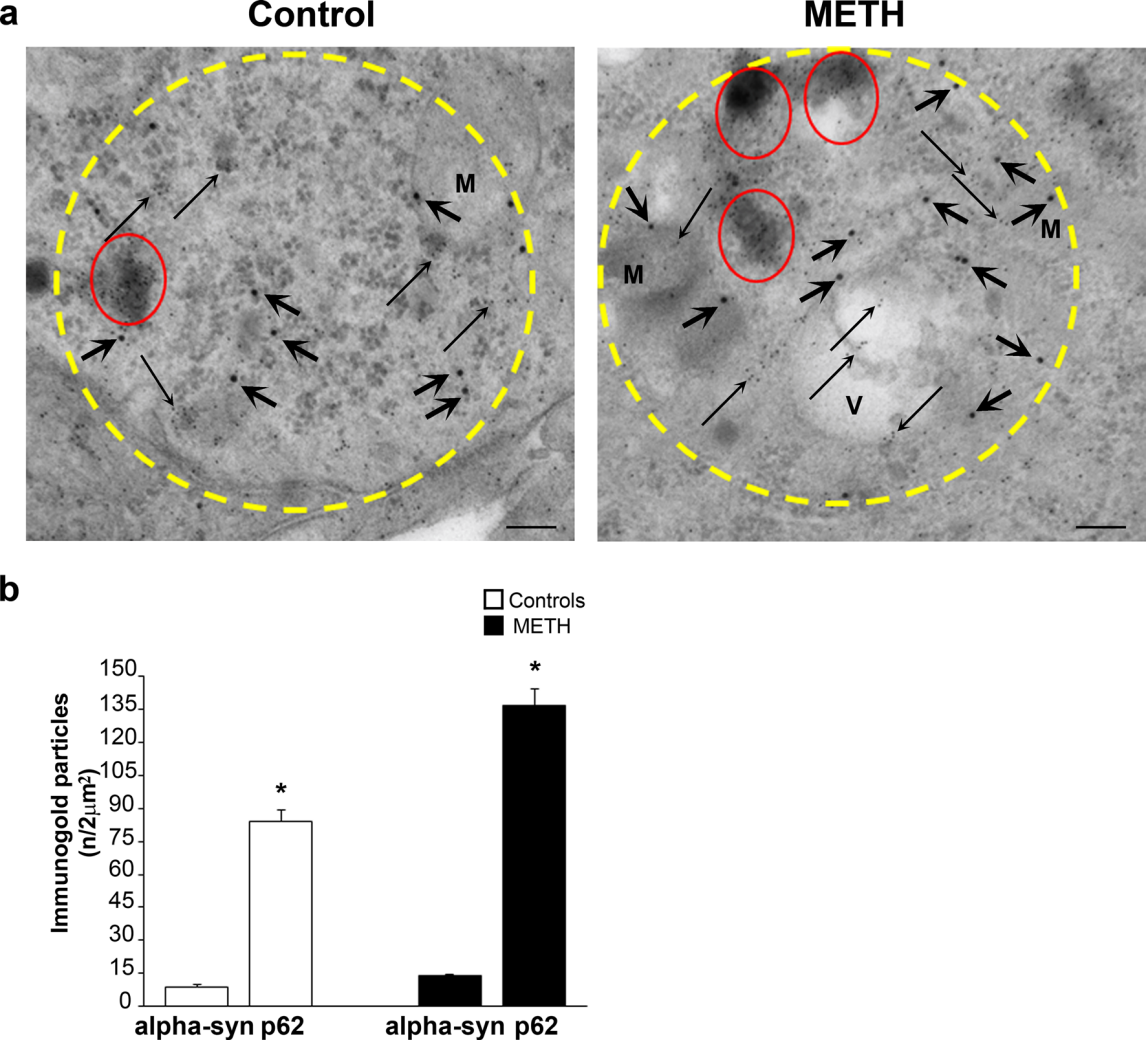
Stoichiometric counts of alpha-syn and p62 proteins within p62-rich regions. Since p62 prevails in alpha-syn-rich regions (previous Fig. 8), where it increases more than alpha-syn following METH, a sampling of cytosolic areas was carried out based on the richest p62 content (see the paragraph Extended Statistics in the Methods section). **a** Representative immuno-electron micrographs show selected p62-rich cytosolic areas (2 µm^2^, yellow dotted circles) from a control cell and a cell treated with METH (100 µM). Again, within these areas selected based on high p62 content, double immuno-gold is carried out against alpha-syn (20 nm immuno-gold particles, shown by thick arrows) and against p62 (10 nm immuno-gold particles, shown by thin arrows). Within these 2 µm^2^ regions rich in p62 content, some domains possess a very dense clusterization of p62, which cannot be indicated due to encumbrance of thin arrows. Therefore, these specific clusters densely packed with p62 immuno-gold particles were indicated by red circles. It is remarkable that the content of vesicular structures within p62-based 2 µm^2^ regions was very abundant following METH. **b** The graph reports the number of immuno-gold particles for p62 and alpha-syn counted within these 2 µm^2^ areas. Within these p62 densely packed clusters, magnification is increased to allow the counting and arrows placement as shown in the representative pictures of next Fig. 10. *M* mitochondria, *V* vesicle. Scale bar: 180 nm. *p < 0.05 compared with controls

**Fig. 10:**
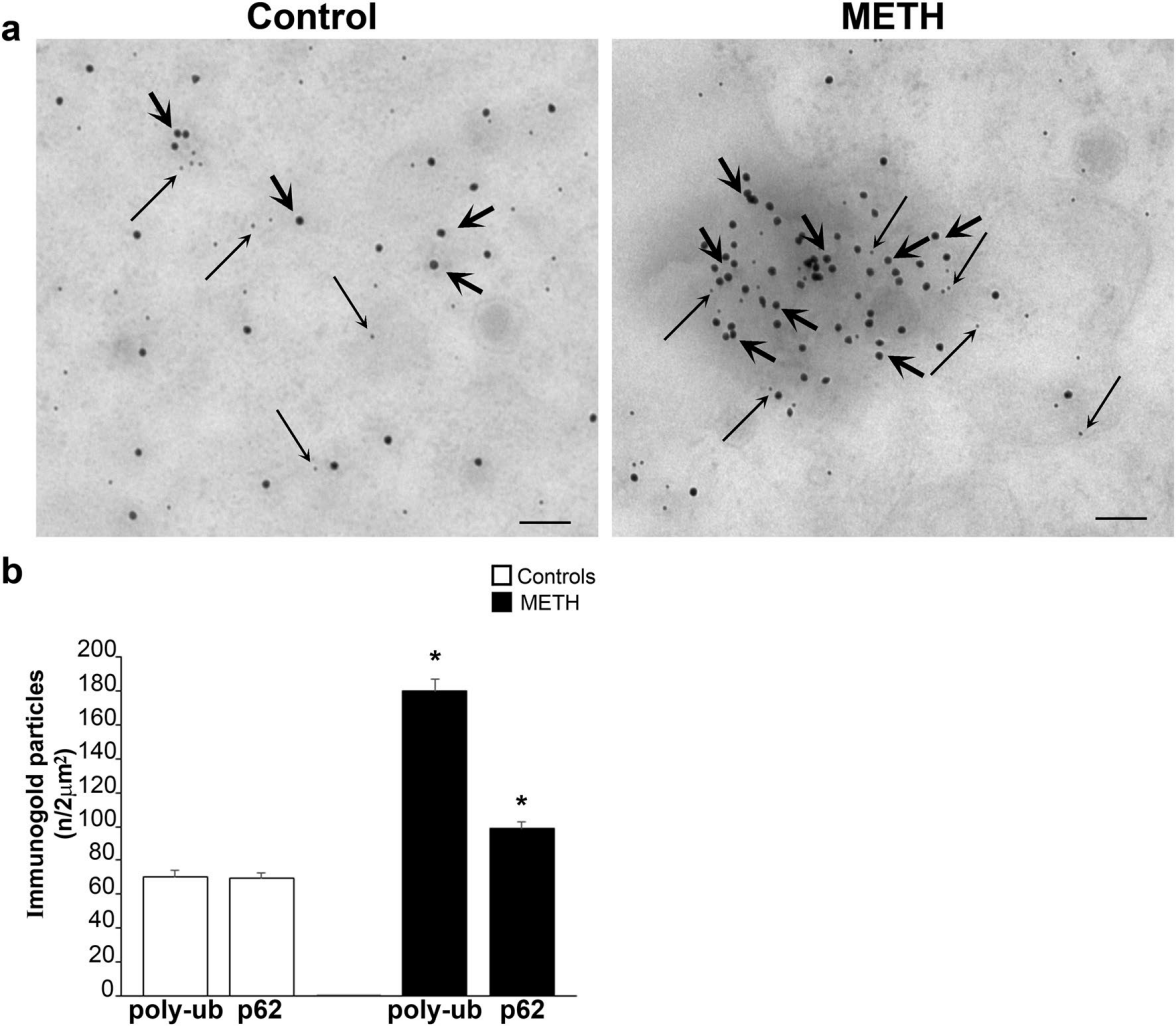
Stoichiometric counts of p62 and poly-ubiquitin proteins within p62-rich regions. When poly-ubiquitin immuno-gold was counted along with p62 within selected 2 µm^2^ areas, in control cells, the amount of poly-ubiquitin was as much abundant as p62. The amount of poly-ubiquitin increased in excess following METH. Due to the high amount of both poly-ubiquitin and p62 proteins within specific clusters found as hot spots within the original 2 µm^2^ areas, a specific analysis was posed on these regions. At this aim, a higher magnification was required to produce representative pictures and discern at best packed immuno-gold particles. In fact, **a** shows at high magnification representative immuno-electron micrographs from a control cell and a cell treated with METH (100 µM). These pictures show the densely packing of immuno-gold against p62 (10 nm, thin arrows) and poly-ubiquitin (20 nm, thick arrows). Although an association between p62 and poly-ubiquitin may be present within control cells, following METH p62 and poly-ubiquitin were tightly associated, which produces dense packages. In this condition, specific domains were evident as represented in the micrograph, where these specific clusters of densely packed p62 + poly-ubiquitin were strictly associated. These very dense domains were measuring an average area of roughly 200 nm, which is tenfold less extended compared with the 2 µm^2^ areas selected for the counts. **b** The graph reports the number of immuno-gold particles for p62 + poly-ubiquitin counted within these areas. It is relevant, when the counts were expressed within the 200 nm densely packed p62 + poly-ubiquitin cell domain, the density of immuno-gold particles increased in excess of tenfold. Again, following METH, an excess of increase in poly-ubiquitin was measured. Scale bar: 108 nm. *p < 0.05 compared with controls

Despite some spatial overlapping between these antigens, electron microscopy indicates that regions possessing the highest p62 content do not necessarily correspond to those regions possessing the highest amount of alpha-syn. This fine detail cannot be appreciated at light microscopy. In fact, when the count was moved from circles owing the highest alpha-syn content to circles selected based on the highest p62 amount, the ratio of p62 compared with alpha-syn approaches tenfold (Fig. 9). In detail as shown in representative Fig. 9a, a similar region (2 µm^2^) with rich vesicles content is shown. In this region, alpha-syn is quite abundant, although the amount of p62 doubles the amount counted in alpha-syn-based selected areas. This indicates that in the course of catecholamine pathology, the amount of proteins, which are key in the structure of inclusions features a number of p62 which is tenfold the amount of alpha-syn specifically counted within those cytosolic spots, which are reminiscent of catecholamine inclusions. The remarkable amount of p62 compared with alpha-syn is similar to the amount of poly-ubiquitin (Fig. 10) and these antigens fully overlap within similar areas as already indicated by immuno-fluorescence of Fig. 6. In fact, the combined immuno-gold staining for p62 and poly-ubiquitin provides the most reliable index of METH-induced pathology, since both antigens increase considerably within the very same vesicular spots. Remarkably, when considering such a combined increase, the difference between control and METH-treated catecholamine cells is striking. In fact, the spatial pattern of p62/poly-ubiquitin spots is structured according to densely packed antigens arranged in a small region (approximately tenfold less extended compared with areas counted so far). A detailed spatial definition of these combined immuno-gold stained areas reveals that moving from alpha-syn richest spots, the size of these gold hallmark for protein accumulation within catecholamine cells needs to be recalculated, since p62 and poly-ubiquitin were placed within similar small cytosolic areas following such a densely packed pattern. These hot spots of densely packed massive immuno-gold particles were roughly tenfold denser within an area, which was roughly tenfold smaller, In fact, the p62/poly-ubiquitin clusters are smaller and they concentrate within an area approaching 200 nm^2^. Thus, the density of p62/poly-ubiquitin molecules is roughly 100-fold higher compared with the density of alpha-syn molecules (counting each antigen in its respective densest spot). It is likely that these hot spots featuring an impressive amount of p62 and poly-ubiquitin density may represent the seeds of neuronal inclusions and/or widespread cytopathology within METH-induced catecholamine cells as already postulated in PD (Sato et al. 2021). The presence of alpha-syn within these hot spots may even be negligible, which witnesses for the relevance of p62 and poly-ubiquitin in seeding protein aggregates in the course of catecholamine cell pathology. Still considering Fig. 10, it is remarkable the occurrence of a short distance between p62 and poly-ubiquitin antigens, which is compatible with the concept of a binding between p62 and poly-ubiquitin chains (as suggested by co-immuno-precipitation, Lazzeri et al. 2018). The striking co-localization of p62 with poly-ubiquitin is fascinating due to the specific physiological role of p62 to shuttle poly-ubiquitinated protein and mitochondria within the autophagosome (Cohen-Kaplan et al. 2016).

Ubiquitin staining is not an issue within these areas, as shown in this manuscript even by immuno-peroxidase and immuno-fluorescence (Fig. 4). This confirms what originally reported when analyzing the structural pathology of catecholamine cells starting from the pioneer data of Iwatsubo et al. (1996) and confirmed by Forno (1996). Therefore, we avoided to produce ubiquitin-based stoichiometry data leaving poly-ubiquitin instead. In fact, as reported in their seminal original study, Iwatsubo et al. (1996) showed that the monoclonal antibody that stained most intensely LBs does not recognize ubiquitin when this is free or within monoubiquitinated aggregates. Conversely, intense immuno-staining is produced by antibodies recognizing poly-ubiquitin chains as well as high-molecular-weight poly-ubiquitinated proteins.

### The plain structure and electron-density of alpha-syn and p62/poly-ubiquitin stained areas

**Fig. 11:**
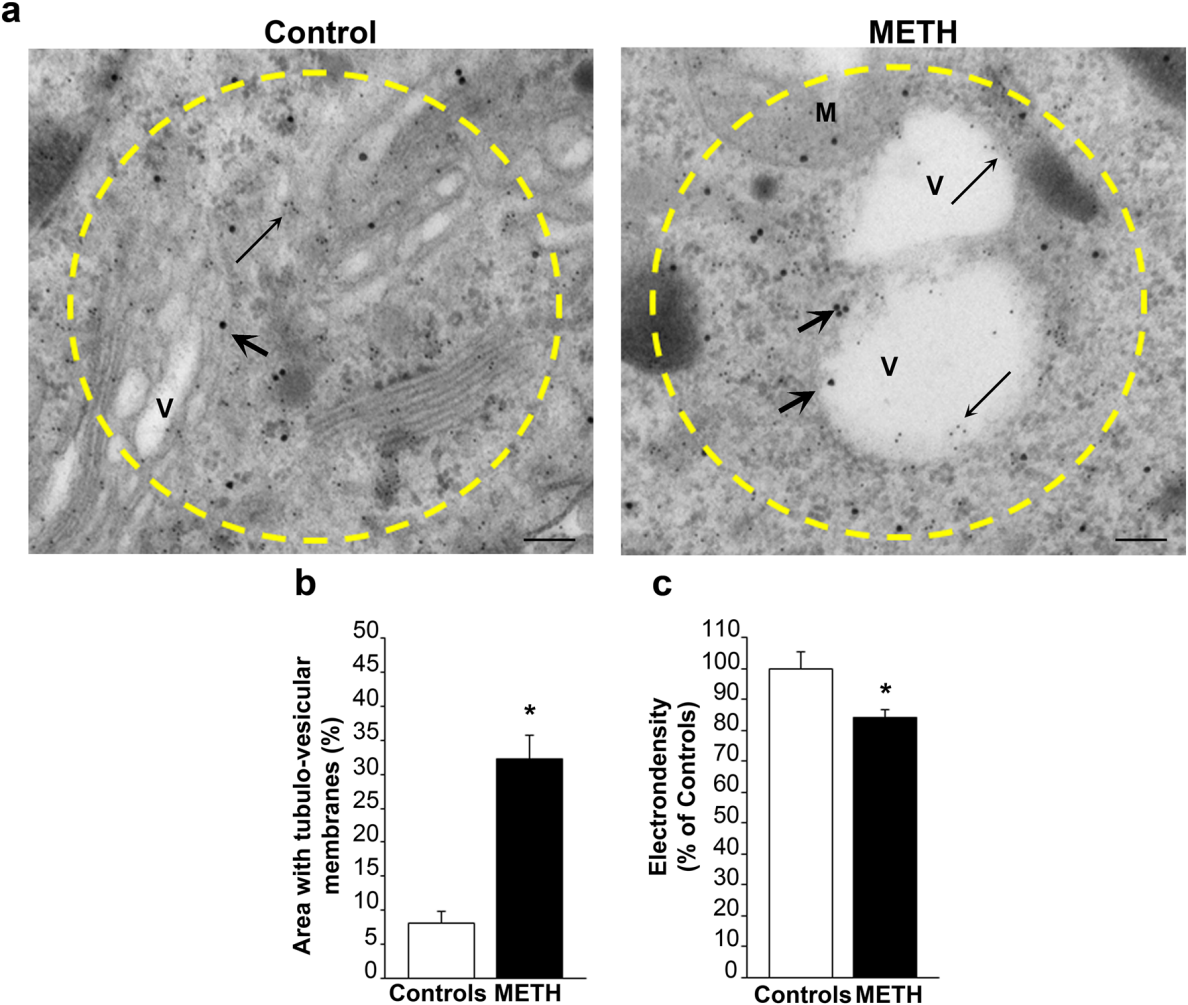
Plain structure and electron density of alpha-syn-rich areas. **a** Representative pictures of alpha-syn-rich 2 µm^2^ areas show a prevalence of vesicles within METH-treated cells compared with the surrounding cytosol and analogous areas from control cells. **b** The graph indicates that the percentage of these vesicular areas was increased following METH (100 µM) compared with controls. **c** The graph reports the electron density of these very same areas measured in controls and following METH (100 µM). In line with the abundance of vesicular structures, these areas own a significant decrease in electron-density. Thick arrows point to alpha-syn immuno-gold particles; thin arrows point to p62 immuno-gold particles. *M* mitochondria, *V* vesicle. Scale bar: 180 nm. *p < 0.05 compared with controls

**Fig. 12:**
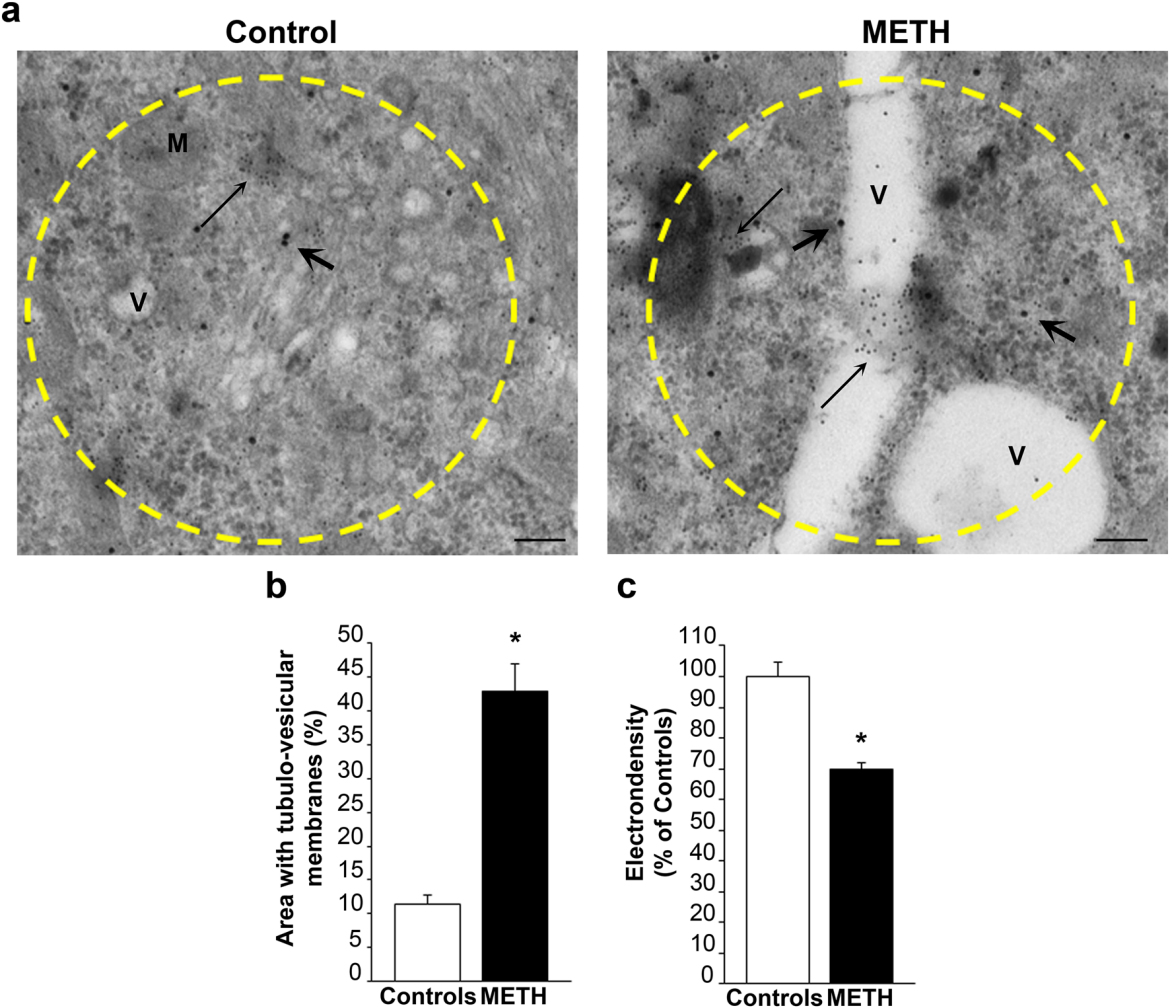
Plain structure and electron density of p62-rich areas. **a** Representative pictures of p62-rich areas show a prevalence of vesicles in the METH-treated cell compared with the control cell. Such a difference is more pronounced compared with that observed within alpha-syn-rich 2 µm^2^ areas reported in Fig. 11a. **b** The graph measures the percentage of vesicle surface within these 2 µm^2^ areas following METH (100 µM), when compared with surrounding cytosol and cytosol from controls. **c** The graph reports the electron density of these very same areas measured in controls and following METH (100 µM). Thick arrows point to alpha-syn immuno-gold particles; thin arrows point to p62 immuno-gold particles. *M* mitochondria, *V* vesicle. Scale bar: 180 nm. *p < 0.05 compared with controls

When considering the structure of these areas densely stained for alpha-syn (representative Fig. 11a), and p62 and poly-ubiquitin (representative Fig. 12a) of METH-treated cells, a prevalence of vesicles either reminiscent of autophagosomes, lysosomes, and mitochondria was present in excess compared with surrounding cytosol as measured in Figs. 11b and 12b. This replicates the qualitative description provided at CLEM by Shahmoradian et al. (2019). This is confirmed by a consistent decrease in electron density within these very same areas, which suggests a lipidic membranous nature forming these tubule-vesicular components for both alpha-syn-defined and p62-defined areas (Figs. 11c, 12c, respectively). In keeping with this, it is not surprising that these areas observed at TEM contain specific key proteins in the neighbor of omegasomes or autophagosome-like structures as shown by Shahmoradian et al. (2019). In fact, in the present study, co-localization of both p62 and poly-ubiquitin often occurs within electron-rare cell domains, where empty vesicles represent the largest areas. In fact, the presence of non-protein structures within these spots of highly immuno-stained cytopathology is quantified here. In detail, within alpha-syn rich regions, 32% of the whole 2 µm^2^ area is taken by electron-rare, presumably mostly lipidic, containing vesicles (Fig. 11b), while in p62/poly-ubiquitin rich areas, this amount rises up to 43% (Fig. 12b). This strongly indicates that the intimate structure of non-protein material covers most of the total pathological region. In summary, these areas are mostly constituted by non-protein components. This is consistent with novel vistas about pathological spots within degenerating catecholamine cells where various organelles and non-protein vesicles are well represented (Iwatsubo et al. 1996; Forno 1996; Shahmoradian et al. 2019; Lashuel 2020; Tang et al. 2021). This confirms an altered shuttling by p62 of poly-ubiquitin bound proteins and mitochondria with the autophagolysosomal compartment, which is in line with the alterations recently described for METH-induced damage (Lazzeri et al. 2018) or in the course of PD (Oh et al. 2022). Moreover, these latter findings are in line with the accumulation of p62, and poly-ubiquitin chain close to mitochondria and autophagolysosomes, which are constantly described in degenerating catecholamine neurons (Nanayakkara et al. 2023).

## Discussion

The present manuscript provides a specific analysis about some key features of cell pathology produced by METH within catecholamine neurons resembling cytopathology, which develops within catecholamine neurons in PD. The detailed analysis of catecholamine cell pathology including inclusions represents a crucial step to understand the neurobiology of diseases such as neurodegenerative disorders affecting catecholamine neurons and the pathology of the addicted brain. A few studies were aimed to characterize these features until the end of the past century. A pioneer study was carried out by Hirsch et al. (1985). The study was designed to purify an antibody specific for LB in PD. Such an approach was followed by the works by Pollanen et al. (1992, 1993, 1994) and the isolation of poly-ubiquitin rather than ubiquitin within LB by Iwatsubo et al. (1996). Soon after, the occurrence of alpha-syn was reported by Spillantini et al. (1997) and such a protein was considered as the major component of LB. The occurrence of alpha-syn is impressive when light microscopy immuno-detection is carried out or when samples are pre-treated with protease and TEM is carried out on protease resistant tissue centrifugates. Nonetheless, at present, no study compared the stoichiometric amount of alpha-syn with other proteins during cytopathology of catecholamine neurons in situ. Therefore, conclusions were largely based on qualitative data. In keeping with such a qualitative analysis, novel approaches using CLEM led to identify an impressive amount of non-protein aggregates within diseased catecholamine neurons, where a number of membranes and vesicular organelles seem to be densely represented (Shahmoradian et al. 2019). Still even in this case, the qualitative approach prevails over data quantification. Therefore, the present study specifically investigated the stoichiometric amount of a few critical proteins including alpha-syn by profiting of an in vitro system administered METH.

Data provided by light microscopy indicate that alpha-syn accumulates dose-dependently following 72 h of METH exposure. This is consistent with data we previously reported in vivo and in vitro, where METH was shown to induce alpha-syn positive inclusions in catecholamine cells of substantia nigra and catecholamine cell lines (Fornai et al. 2004). Remarkably, as reported here, other key antigens involved in the degeneration of catecholamine-containing neurons such as p62 and poly-ubiquitin increase according to a roughly similar dose–response curve. In keeping with cell pathology of catecholamine neurons, the occurrence of poly-ubiquitin appears to be way more relevant than ubiquitin alone as reported by pioneer works (Iwatsubo et al. 1996). In the present manuscript, evidence is provided that dose-dependent increase in alpha-syn immuno-fluorescence is shifted to the left compared with dose-dependent METH-induced cell death. The latter was assessed by three independent methods (H&E, TB, FJB) which given similar results, thus providing an internal validation across various techniques. In particular, the strong effects obtained following FJB histochemistry indicate such a marker as a reliable tool to assess cell degeneration even in vitro following METH. Remarkably, cell damage assessed by FJB histochemistry is slightly more dramatic compared with H&E and TB. As briefly mentioned in the results section, this may also depend on the specific targets of FJB, which may stain molecules other than those strictly involved in cell degeneration and still induced by METH exposure such as specific poly-amines.

Immuno-fluorescence and immuno-peroxidase carried out following a dose of METH selected to increase key proteins in the absence of frank cell death indicate a marked increase of alpha-syn. The METH-induced increase in alpha-syn is concomitant with an increase of p62 and poly-ubiquitin. When roughly assessed at densitometry (both immuno-peroxidase and immuno-fluorescence), the increase of p62 and poly-ubiquitin in METH-treated cells is roughly twofold compared with the increase in alpha-syn.

In the second part of the study, light microscopy was matched with ultrastructural stoichiometry through an experimental approach combining light and electron microscopy (CLEM) as recently applied, to provide a qualitative analysis of the intimate structure of alpha-syn-positive LB in catecholamine neurons by Shahmoradian et al. (2019). Such an approach was used here by starting from the identification of pale eosinophilic areas identified at H&E, to focus on specific regions where semi-thin toluidine blue-stained sections confirm that pale cytosol areas were stained by immuno-peroxidase for alpha-syn or p62. Within these selected cell spots, immuno-gold and plain electron microscopy allowed to dissect by stoichiometry quantitative amount of specific proteins occurring following METH administration. Similarly, the fine ultrastructure of cell regions possessing a high amount of these proteins was assessed. In keeping with recent data about cell pathology of catecholamine neurons such as LB from PD patients, here, we found an impressive amount of membranous vesicles within critical spots either staining for alpha-syn and/or p62. It was remarkable that these cell regions possess an amount of p62 molecules within 2 µm^2^ areas surpassing at large (tenfolds) the amount of alpha-syn counted within areas of the same size. Again, the increase in p62 was concomitant with poly-ubiquitin. In fact, these antigens where densely packed providing a cell domain, which indeed was clustered within smaller regions (200 nm^2^) compared with those featuring increased alpha-syn immuno-staining. This indicates that the amounts of p62/poly-ubiquitin molecules within specific clusters reach a density of roughly 100-fold compared with the density of alpha-syn. The occurrence of high amounts of p62/poly-ubiquitin way in excess compared with alpha-syn was quite impressive when comparing immuno-gold stoichiometry (100-fold) with rough immuno-fluorescence densitometry (twofold). The size of these p62/poly-ubiquitin-rich clusters was much smaller compared with the scattered clusters of alpha-syn. Again, the relationship of p62/poly-ubiquitin with abnormal amounts of membranous organelles surpasses the association between organelles and alpha-syn. These data indicate the quantitative composition of specific protein and non-protein content within cell domains during METH-induced pathology. The present data also emphasize the different information between semi-quantitative densitometry roughly esteemed at light microscopy and the stoichiometry quantification of authentic protein amounts and tubulo-vesicular areas detected at TEM. This strong discrepancy explains technical and conceptual uncertainties in the definition of catecholamine cell pathology. This is likely to rely on the non-linear relationship between fluorescence/peroxidase signal and protein amount compared with specificity of immuno-gold stoichiometry. A limit of the present data, which needs to be taken into account concerns the administration of METH within catecholamine cell cultures. Nonetheless, there is a remarkable consistency with data obtained ex vivo within nigral catecholamine neurons of transgenic mice expressing PD-inducing genes, which were recently reported to sort similar results. In fact, the occurrence of p62 and poly-ubiquitin appears within LB-like bodies at early time intervals (2 months), while alpha-syn joins the inclusions only at 9 months of age. These led the authors to suggest that the seeding of pathological inclusions may be represented by p62 rather than alpha-syn (Kurosawa et al. 2016; Noda et al. 2022; Sato et al. 2018, 2020, 2021). The occurrence of abundant lipid-containing membranous structure, focally, close to the p62 and poly-ubiquitin clusters and to alpha-syn molecules matches the qualitative data recently obtained by Shahmoradian et al. (2019) concerning the cell pathology of nigral neurons from PD patients, where the composition of LB was extremely variable and rich in lipid membranes and various organelles.

This is quite remarkable and indeed unexpected considering that alpha-syn is indicated as the major component of a number of neuronal inclusions. Such a discrepancy may depend on the use of METH or the specific cell line. Again, technical differences between light and electron microscopy may further explain a number of issues which affect the semi-quantitative measurement of immuno-staining compared with stoichiometry measurement at TEM. All these issues may contribute to decipher why, despite the amount of alpha-syn stoichiometrically measured is 100-fold less dense, the fluorescence or peroxidase is very intense; for instance: (1) the close space between p62 and poly-ubiquitin proteins does not allow an easy access to peroxidase- or fluorophores-conjugated antibodies; (2) the binding sites of p62 and poly-ubiquitin are overwhelmed by an excess of endogenous protein substrates competing with primary and secondary antibodies; (3) the co-chaperonine nature of alpha-syn may provide multiple binding sites, which in turn may attract a high number of primary antibodies; (4) the baseline recruitment and buffering of p62 and poly-ubiquitin is elevated even in baseline conditions due to a powerful engagement of both these molecules; (5) the recruitment of alpha-syn in baseline conditions by endogenous proteins is much less intense; (6) in addition, the density of alpha-syn may produce an optimal distance to detect a strong signal-to-noise ratio for the fluorophore/peroxidase bound to secondary antibodies. All these issues potentially altering the authentic protein amount are erased by TEM stoichiometry. In fact, a main focus of the present study is to provide the authentic protein amount by in situ protein measurement (Bergersen et al. 2008). Still, even TEM detection may introduce other bias such as different access to antigens of immuno-gold particles depending on their size due to mutual steric encumbrance, where larger immuno-gold particles are underscored compared with smaller particles, which may lead either to mask or unmask double staining. Such an issue was approached by reverting the size of immuno-gold particles binding the primary antibodies. When the size of immuno-gold used to stain two antigens was switched, the data were confirmed. In fact, the authentic amount of p62, and alpha-syn did not vary when the size of immuno-gold particles was reverted. One major outcome of the study is the demonstration that p62/poly-ubiquitin, apart from being more abundant in baseline conditions compared with alpha-syn, undergo an upregulation and clusterization following METH, which surpasses at large the increase, which is measured for alpha-syn. This clusterization occurs preferentially as densely packed clusters within electron-rare cytosolic regions, where the ultrastructure reveals a high tubulo-vesicular component mainly made up of autophagosomes/omegasomes and mitochondria. This is in line with the physiological role of p62, which acts as a shuttle to import protein and other substrates including mitochondria through a poly-ubiquitin chain within the nascent autophagosome (Cohen-Kaplan et al. 2016). Thus, the accumulation of p62/poly-ubiquitin is supposed to occur where an impairment of the autophago-lysosome system takes place. This is in line with the molecular effects of METH (Lazzeri et al. 2018) and it is strikingly similar to the impairment of these organelles as emerging in recent studies in PD patients. This explanation inherently addresses the second main finding of the present work which revels the paucity of protein compared with membranous organelles to build up the fine neuropathology induced by METH within catecholamine neurons. Since this occurs also in PD brain, it is not surprising that the mimicking of parkinsonian neuropathology produced by METH reproduces quite similar findings. Of course, from a methodological stand point, we are obliged to leave the benefit of the doubt concerning a profound diversity between LB and METH-induced inclusions, although some literature tend to overlap these conditions (Kousik et al. 2014). In any case, this fully applies to the pathology of catecholamine neurons in the addicted brain, where a long-term METH abuse predisposes to develop PD (Kousik et al. 2014; Lappin and Darke 2021; Rumpf et al. 2017; Shin et al. 2021; Vincent and Shukla 2023).

The advantage of the present study relies on applying CLEM in vitro where the high reproducibility and flexibility of the model allows to count single protein molecules. In fact, previously, CLEM was used ex vivo to analyze qualitatively the structure of LB without assessing the amount of specific proteins by immuno-gold. In these conditions, it is difficult to preserve antigen conformation within human post-mortem tissue. However, in 1996, immuno-gold was used to measure ubiquitin in post-mortem human brain tissue. Moreover, the immuno-gold was not used to quantify the antigen and it was not carried out in situ within specific cell domain, since it was used in cell fractions following centrifugation in sucrose gradient (Iwatsubo et al. 1996). Again, this study was carried out before alpha-syn was recognized as a marker of neuropathology, and at that time, p62 was not explored in neurodegeneration. Therefore, this pioneer study remained seminal to emphasize the poly-ubiquitin rather than the ubiquitin staining of PD inclusions leaving unsolved the amount of p62 and alpha-syn. The present manuscript shed light on a primary role of p62 in METH toxicity, which is reciprocated by recent literature obtained from mice models expressing a number of genes inducing PD (Kurosawa et al. 2016; Noda et al. 2022; Sato et al. 2018, 2020, 2021). These data show that p62 and poly-ubiquitin appears within LB-like bodies at early time intervals (2 months) while alpha-syn joins the inclusions only at 9 months of age. This is fascinating in the light of recent findings obtained in a variety of experimental condition including PD brain, which show how a primary dysfunction of p62 leads to autophagy inhibition and subsequent alpha-syn accumulation and secretion and PD spreading (Oh et al. 2022).

## Concluding remarks

This combined representation of METH-induced pathology encompasses the measurement in situ of alpha-syn, p62, and poly-ubiquitin within their respective densest spots, where membranous organelles are abundant. From this study it appears that, when translating immuno-detection from light into electron microscopy, the nature of cell pathology and pathological inclusions is profoundly modified. In fact, the pathological features observed at immuno-peroxidase and immuno-fluorescence, which may lead to a clear-cut definition of abnormal and physiological compartments are much less evident. When searched at TEM, a pathological spot representing an inclusion at light microscopy may not be evident and the shape, contour, composition of an alpha-syn-rich area and the cytosol can appear under a pleomorphic condition. The increase of magnification and the use of an electronic beam reveal a different scenario with abundant occurrence of a number of membrane bound organelles which may vary in shape and composition. This is in sharp contrast with the apparent clear-cut which is produced by immuno-peroxidase and immuno-fluorescence used to detect alpha-syn at light microscopy. In this case, typical shapes appear which cannot be replicated at ultrastructural level. Contrariwise, when other proteins are analyzed in depth at high magnification, specific areas can be defined. This is the case of p62, which provides dense protein clusters at ultrastructural stoichiometry prevailing at large over the amount of alpha-syn. The discrepancy between light and electron microscopy mostly when referring to alpha-syn may be further explained by the natural trend of this protein to aggregate. In fact, seminal articles indicate that roughly 30% of native alpha-syn spontaneously aggregate and binding other proteins (Burré et al. 2013, 2014). During such a process, the native protein may produce a spontaneous inter-conversion of protein structure between alpha-helical and beta-strands (Sirangelo et al. 2002). In turn, these effects may alter the access and binding of modified protein domains to secondary antibodies. This is likely to affect the diffusion of immuno-peroxidase and immuno-fluorescence compared with the encumbrance and binding of antigens with immuno-gold particles. This issue does not seem to be very likely to affect the immuno-gold staining, since the shift from small into large immuno-gold particles does not affect the results. It is more likely that the protein aggregation, and shifting in secondary protein structure, which occurs for alpha-syn may enhance the non-stoichiometric staining obtained at immuno-peroxidase or immuno-fluorescence.

### Supplementary Information

Below is the link to the electronic supplementary material.

Supplementary file1 (PDF 182 kb)

## Data Availability

The data that support the findings of this study are available from the corresponding author upon reasonable request.
