# Supplementary material for: Combined light and electron microscopy (CLEM) to quantify methamphetamine-induced alpha-synuclein-related pathology
Source: J Neural Transm (Vienna). 2024 Feb 17;131(4):335–58. doi: 10.1007/s00702-024-02741-x (PMC11016004; doi:10.1007/s00702-024-02741-x)
Supplement: Supplementary file 1 — Supplementary file1 (PDF 182 kb) [file 702_2024_2741_MOESM1_ESM.pdf]

Journal of Neural Transmission

Combined light and electron microscopy (CLEM) to quantify methamphetamine-induced alpha-synuclein related pathology

Michela Ferrucci[1,§], Paola Lenzi[1,§], Gloria Lazzeri[1], Carla L. Busceti[2], Alessandro Frati[2,3], Stefano Puglisi-Allegra[2], Francesco Fornai[1,2,*]

[1]Department of Translational Research and New Technologies in Medicine and Surgery, University of Pisa, Via Roma 55, 56126 Pisa, Italy. michela.ferrucci@unipi.it; paola.lenzi@unipi.it; gloria.lazzeri@unipi.it; francesco.fornai@unipi.it.

[2]Istituto di Ricovero e Cura a Carattere Scientifico (I.R.C.C.S.) Neuromed, Via Atinense 18, 86077 Pozzilli, Italy. carla.busceti@neuromed.it; stefano-puglisiallegra@neuromed.it; francesco.fornai@neuromed.it.

[3]Neurosurgery Division, Human Neurosciences Department, Sapienza University, 00135 Rome, Italy. alessandro.frati@uniroma1.it.

[§]These Authors contributed equally to the present work
*Corresponding Author:
Francesco Fornai,
francesco.fornai@neuromed.it;
francesco.fornai@unipi.it.

ORCID of the authors:
Michela Ferrucci: 0000-0002-3009-7160; Paola Lenzi: 0000-0002-4734-8798; Gloria Lazzeri: 0000-0003-4607-098X; Carla L. Busceti: 0000-0002-1417-1893; Alessandro Frati: not available; Stefano Puglisi-Allegra: 0000-0002-9433-2000; Francesco Fornai: 0000-0002-3883-5084.

Comments on data variability of METH-induced cell damage

For instance the amount of severe cell damage assessed by H&E is based on actual lack of cell structures (unique among light microscopy procedures) and the presence of remarkable alterations of cell shape and size and a faint cytosol staining or dense nuclear staining frankly differing from standard control cells. In this case, the authentic count carried out in control cultures is ruling out the unavoidable cell degeneration, which occurs also in control conditions. This latter feature may lead to overestimate the effects of METH toxicity since natural ongoing toxicity is cut off. Again, in keeping with H&E staining, the amount of cell alterations required to call a cell out of the viable range needs to be severe and to be overtly evident at light microscopy, which may conversely reduce the count of irreversible cell damage when finely detected at sub-cellular level. In the case of FJB, we still keep a fideistic approach since we still lack an in-depth knowledge of which and how many markers are responsible for FJB-induced histofluorescence. In this case, additional bias may lead to the occurrence of METH-derived, though non-damage dependent compounds, which target FJB enhancing the fluorescent signal. This may explain why the count following FJB is the highest compared with all other methods. Even the time window is crucial since variations of FJB fluorescence depends on the onset, persistence and decay of the fluorescent dye. In fact, it has been reported that the gold standard time window to measure FJB fluorescence is placed between 24 and 72 hours although this time interval does not provide steady fluorescence and a linear correlation is lacking. For the same reason the mechanism of toxicity may mask the very same molecules, thus hiding a number of degenerating cells or attenuating their fluorescent signaling.
